# Vivid COVID-19 LAMP is an ultrasensitive, quadruplexed test using LNA-modified primers and a zinc ion and 5-Br-PAPS colorimetric detection system

**DOI:** 10.1038/s42003-023-04612-9

**Published:** 2023-03-02

**Authors:** Adrián Szobi, Katarína Buranovská, Nina Vojtaššáková, Daniel Lovíšek, Halil Önder Özbaşak, Sandra Szeibeczederová, Liudmyla Kapustian, Zuzana Hudáčová, Viera Kováčová, Diana Drobná, Piotr Putaj, Stanislava Bírová, Ivana Čirková, Martin Čarnecký, Peter Kilián, Peter Jurkáček, Viktória Čabanová, Kristína Boršová, Monika Sláviková, Veronika Vaňová, Boris Klempa, Pavol Čekan, Evan D. Paul

**Affiliations:** 1 MultiplexDX, s.r.o., Comenius University Science Park, Ilkovičova 8, 841 04 Bratislava, Slovakia; 2 MultiplexDX, Inc., One Research Court, Suite 450, Rockville, MD 20850 USA; 3 University of Cologne, Institute for Biological Physics, Zülpicher Str. 77, 50937 Köln, Germany; 4 AstonITM s.r.o., Račianska 153, 831 54 Bratislava, Slovakia; 5 Biomedical Research Center, Slovak Academy of Sciences, Institute of Virology, Dúbravská cesta 9, 845 05 Bratislava, Slovakia; 6 Department of Microbiology and Virology, Faculty of Natural Sciences, Comenius University, Ilkovičova 6, 842 15 Bratislava, Slovakia; 7 Present Address: Stanford University, 730 Escondido Rd., Stanford, CA 94305 USA

**Keywords:** Assay systems, Diagnostic markers

## Abstract

Sensitive and rapid point-of-care assays have been crucial in the global response to SARS-CoV-2. Loop-mediated isothermal amplification (LAMP) has emerged as an important diagnostic tool given its simplicity and minimal equipment requirements, although limitations exist regarding sensitivity and the methods used to detect reaction products. We describe the development of Vivid COVID-19 LAMP, which leverages a metallochromic detection system utilizing zinc ions and a zinc sensor, 5-Br-PAPS, to circumvent the limitations of classic detection systems dependent on pH indicators or magnesium chelators. We make important strides in improving RT-LAMP sensitivity by establishing principles for using LNA-modified LAMP primers, multiplexing, and conducting extensive optimizations of reaction parameters. To enable point-of-care testing, we introduce a rapid sample inactivation procedure without RNA extraction that is compatible with self-collected, non-invasive gargle samples. Our quadruplexed assay (targeting E, N, ORF1a, and RdRP) reliably detects 1 RNA copy/µl of sample (=8 copies/reaction) from extracted RNA and 2 RNA copies/µl of sample (=16 copies/reaction) directly from gargle samples, making it one of the most sensitive RT-LAMP tests and even comparable to RT-qPCR. Additionally, we demonstrate a self-contained, mobile version of our assay in a variety of high-throughput field testing scenarios on nearly 9,000 crude gargle samples. Vivid COVID-19 LAMP can be an important asset for the endemic phase of COVID-19 as well as preparing for future pandemics.

## Abstract (web summary)

Vivid COVID-19 LAMP is an ultrasensitive zinc ion and zinc sensor based colorimetric detection system, reliably detecting viral RNA from self-collected, non-invasive gargle samples for rapid point-of-care testing.

## Introduction

To combat the next phase of the global coronavirus disease 2019 (COVID-19) pandemic and prepare for future outbreaks, scalable diagnostic methods that are easily deployed to point-of-care testing (POCT) locations are critical. Rapid antigen tests (RATs), which detect viral proteins, dominate POCT due to their simplicity and rapid results. However, the lower sensitivity of RATs and therefore greater risk of false-negative results is the main limitation^1–4^. Although RT-qPCR remains the gold standard for COVID-19 diagnosis, its relative technical complexity precludes on-demand testing and timely results. Nucleic acid amplification tests (NAATs) based on reverse-transcription loop-mediated isothermal amplification (RT-LAMP) is a viable alternative, since the method is rapid, requires minimal laboratory equipment, can be performed by personnel without advanced lab training, and has sensitivity approaching that of RT-qPCR^5–10^.

The LAMP reaction contains four to six primers responsible for creating a dumbbell-like structure that contains self-priming sites for a strand-displacement DNA polymerase^11,12^. Compared to RT-qPCR, RT-LAMP amplifies the product at constant temperature (usually 60–65 °C), provides quicker results (<1 h), and can be used without prior RNA extraction due to the enzymes robustness against classical PCR inhibitors. RT-LAMP produces considerably more amplicon than PCR, and a variety of techniques have been developed to detect amplicons including fluorescent detection (e.g., fluorogenic probes^13–16^, intercalating dyes^17–21^, and flourescently-labeled primers^20,22^), oligonucleotide-functionalized plasmonic sensors^23,24^, lateral flow biosensors^25,26^, CRISPR-based detection systems^27–36^, and sequencing^22,28,37^. Alternatively, LAMP amplification by-products (e.g., pyrophosphate and hydrogen ions) can be detected using colorimetric indicators broadly divided into those that are pH-independent such as Calcein^38,39^, Hydroxynaphthol Blue (HNB)^40,41^, and Leuco Crystal Violet^42–44^ as well as pH-dependent indicator dyes like Phenol Red^21,27,45–59^. Although these colorimetric detection systems permit visualization of reaction results by the naked eye, they have important limitations. Calcein indicators require the addition of manganese ions, which are inhibitory to amplification and can induce random mutagenesis, while other indicators (e.g., HNB) that are dependent on magnesium ions (Mg^2+^) prevent independent optimization of detection and amplification reaction as both are tied to the concentration of the same ion. Some also require a UV-illuminator or turbidimeter due to difficulties to distinguish a subtle color change. The pH-based colorimetric indicators all require weakly buffered conditions^45^; thus, in the absence of an RNA extraction step, patient samples that contain variable pH or buffering conditions present challenges for reliable detection. Indeed, during the COVID-19 pandemic, there have been a plethora of reports about spurious color changes that arise when using pH-based LAMP reaction^55–59^ with many quick fix solutions.

Here, we present the Vivid COVID-19 LAMP diagnostic assay, a rapid, ultrasensitive, quadruplexed test that uses a metallochromic-based colorimetric detection system with the colorimetric approach based on a 5-Br-PAPS (2-(5-Bromo-2-pyridylazo)-5-[N-propyl-N-(3-sulfopropyl)amino]phenol) indicator that forms a complex with zinc ions (Zn^2+^) giving the solution a bright magenta color. Pyrophosphates produced during DNA amplification displace Zn^2+^ from the Zn^2+^/5-Br-PAPS complex shifting the color from magenta to yellow so that reaction results are easily discernible. Importantly, since the 5-Br-PAPS detection system complexes with Zn^2+^, it is independent of pH and Mg^2+^ concentration. In combination with our custom-developed reagents for direct sample inactivation of crude gargle samples, it resolves the limitations of classical metallochromic, and pH-based detection methods and it is especially well suited for direct, RNA extraction-free RT-LAMP. By incorporating locked nucleic acids (LNAs) in select LAMP primers of our quadruplexed reaction (targeting E, N, ORF1a, and RdRP) and conducting extensive optimization of our inactivation buffer, master mix composition, and reaction parameters, we developed an RT-LAMP assay with excellent sensitivity and clinical performance using both extracted RNA and unprocessed gargle and nasopharyngeal (NP) swab specimens. With a rapid, streamlined workflow of ~50 min, RNA extraction-free format, and sensitivity that rivals even the best RT-qPCR tests, our Vivid COVID-19 LAMP test is ideal for POCT. We demonstrate the utility of our Vivid COVID-19 LAMP test in a variety of POCT scenarios that included conducting nearly 9,000 LAMP tests.

## Results

### LNA-modified LAMP primers enhance reaction sensitivity and speed in a primer-specific manner

**Fig. 1:**
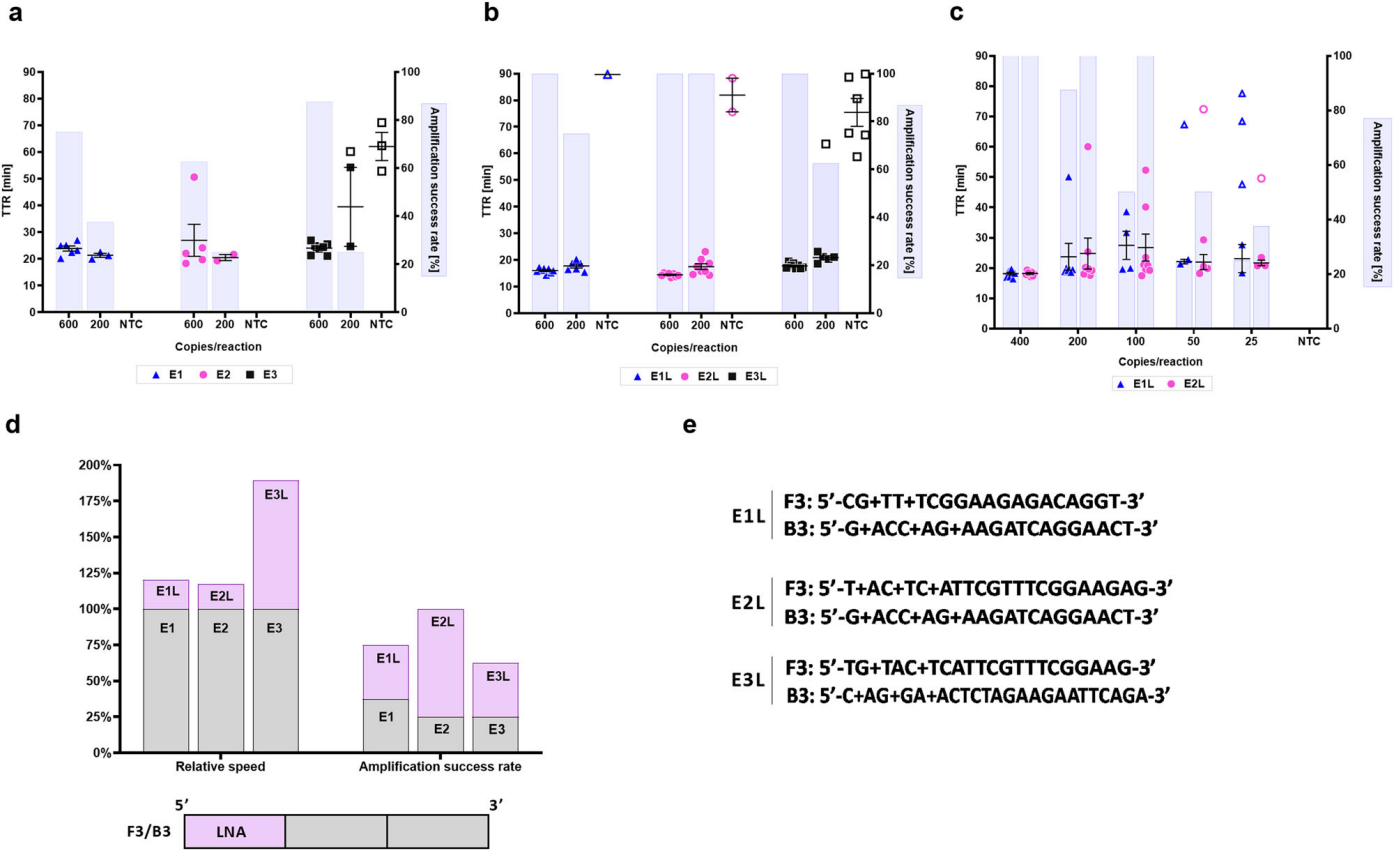
Primer set screening for the detection of SARS-CoV-2 E gene. **a** Performance, measured as TTR, of the top three primer set candidates (E1, E2, E3) for SARS-CoV-2 E gene at the indicated number of synthetic SARS-CoV-2 RNA copies per reaction. **b** Comparison of E1, E2, and E3 primer sets containing LNA modifications near the 5’ end of their F3/B3 outer primers, labeled as E1L, E2L, E3L. Reaction conditions are identical with **a**. **c** An extended comparison of limits of detection of sets E1L and E2L. **d** Amplification improvements obtained by LNA modifications in all three primer set candidates displayed as changes in relative speed and higher amplification success rate. Based on data from **a** and **b** at 200 cp/rxn. Relative speed is mean TTR of base set divided by TTR of LNA-modified set. **e** Positions of LNA bases in sequences of F3/B3 outer primers in primer sets E1L, E2L, and E3L. Open symbols represent non-specific products, whereas closed symbols are specific as determined by melt curve analysis of amplification products. The highest time to reaction value on y-axis signifies the total duration of the reaction. Amplification success rate shows the number of samples that amplified over the course of the reaction. For NTC reactions, PCR-grade water was used as the input. All experiments were performed using “NEB WarmStart” reaction mix. “+N” signifies the presence of an LNA base. Error bars represent standard error of the mean. In all experiments, *n* = 8 technical replicates per group were used. TTR time to reaction, NTC no-template control.

We selected the envelope (E) gene as our first target to design LAMP primers because it is conserved, located towards the 3’ end of the SARS-CoV-2 genome, and its transcripts are enriched in subgenomic RNA^60^. Using PrimerExplorer V5, we designed 3 E gene primer sets named E1, E2, and E3 (Supplementary Data 2) and tested them with New England Biolab’s WarmStart LAMP kit supplemented with the SYTO 9 dye, allowing us to detect amplification by both acidification-induced color change and real-time quantitative fluorescence. Initial tests revealed subpar analytical sensitivity approaching 600 copies/reaction as well as sporadic nonspecific amplification in the E3 set (Fig. 1a). Since the SARS-CoV-2 genome, as well as other human pathogenic coronaviruses, is AU rich^61^, this leads to challenges in designing LAMP primers with high enough *T*_*m*_ to be compatible with strand-displacing Bst polymerases. We sought to improve LAMP sensitivity by introducing LNA-modified bases to increase primer *T*_*m*_, a common approach in PCR to improve target recognition and primer-template dissociation kinetics^62,63^.

We introduced LNAs near the 5’ end of all LAMP primers (in case of inner primers in both F2/B2 and F1c/B1c) to increase the *T*_*m*_ to match the recommended *T*_*m*_ for templates with normal (40–60%) AT(U) content (Supplementary Data 2). Unexpectedly, increasing *T*_*m*_ of inner primers (both regions together or separately) and loop primers reduced speed and sensitivity and promoted non-specific amplification – the only exception was BIP/B1c in the E1L/E3L primer set (Supplementary Fig. 1); however, introduction of LNAs into the F3/B3 outer primers resulted in marked gains in assay sensitivity and speed for all 3 primer sets labeled E1L, E2L, and E3L, with the E2L primer set detecting all replicates at 200 copies/reaction with a concomitant reduction in time to reaction (Fig. 1b–e). An LoD test (Fig. 1c) of the two more promising sets, E1L and E2L, showed reasonable performance down to 100 and 50 copies/reaction, respectively.

### Optimization of reaction conditions and multiplexing improves assay performance

**Fig. 2:**
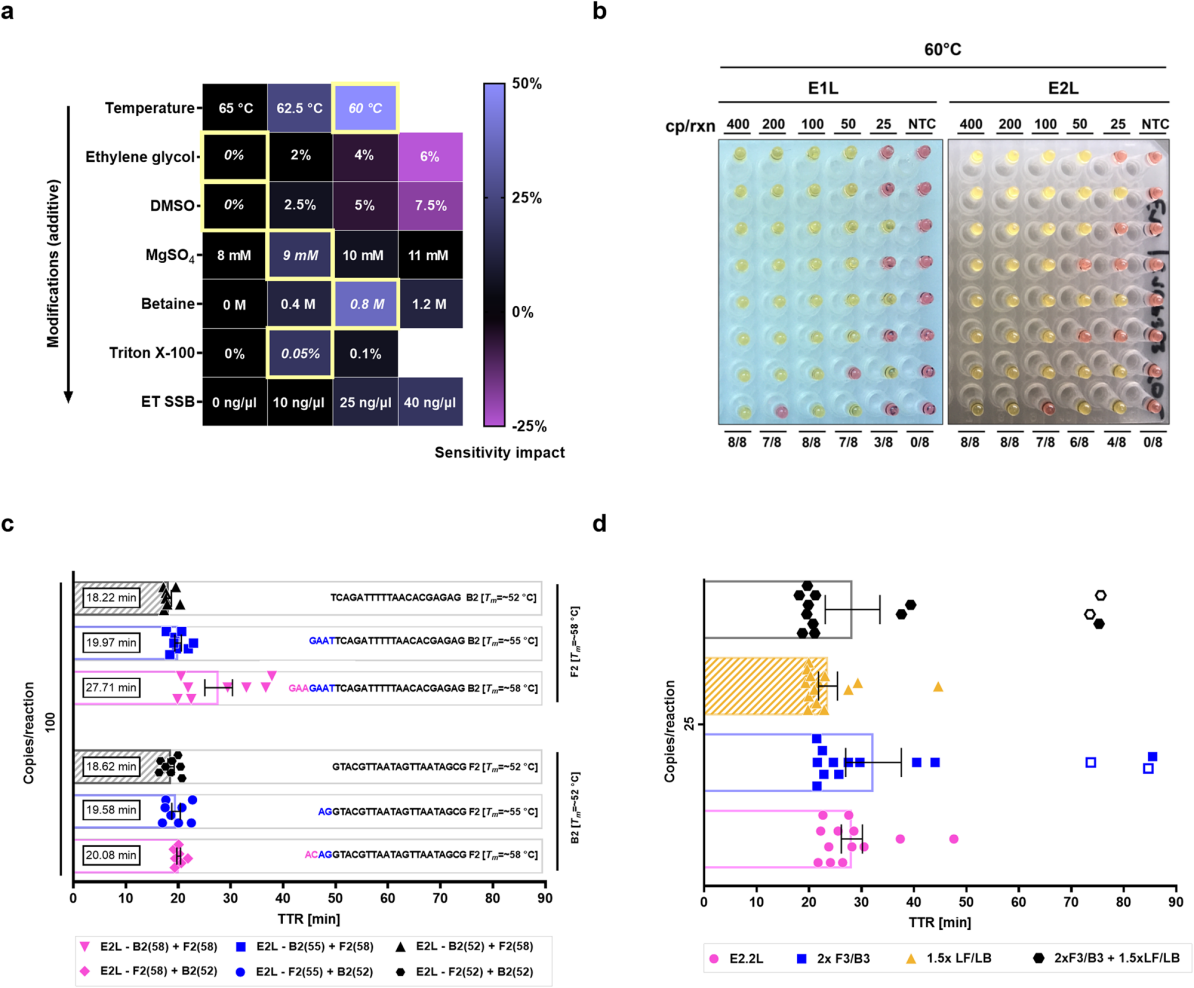
Reaction optimization and primer set modification for the detection of SARS-CoV-2 E gene. **a** Heat map showing the impact of master mix modifications on reaction sensitivity with the E2L primer set. The sensitivity impact represents the change in reaction sensitivity, which was evaluated as percent change in amplification success rate at 25 copies/reaction with 16 replicates per condition. All reactions were run for 60 min. Rectangles highlighted in yellow show parameters carried over to the next phase of testing; therefore, modifications are additive going from top to bottom. **b** Comparison of E1L and E2L primer set sensitivity at 60 °C measured as number of positive replicates. All reactions were run for 60 min and were evaluated by colorimetric change only. **c** Effect of *T*_*m*_ of F2/B2 priming regions in FIP/BIP primers on the performance of the E2L primer set. Primers were adjusted by adding or removing complementary bases towards the 5’ end of F2 or B2 priming regions in such a way that the *T*_*m*_ differential between 2 sequential variants was ~3 °C (as predicted by PrimerExplorer V5). The best performing priming regions have patterned bars. **d** Outcome of increased auxiliary primer concentrations. The graph shows identical reactions differing only in the concentration of outer primers (F3/B3), loop primers (LF/LB), or both. The best performing modification has a patterned bar. Open symbols represent non-specific products, whereas closed symbols are specific as determined by melt curve analysis of amplification products. The highest time to reaction value on x-axis signifies the total duration of the reaction. For NTC reactions, PCR-grade water was used as the input. All experiments in **a** were performed using “NEB WarmStart” reaction mix with additional specified additives while for **b**–**d** “NEB WarmStart 1.1” reaction mix was used. Error bars represent standard error of the mean. In all experiments, *n* = 8 technical replicates per group were used. TTR time to reaction, NTC no-template control, ET SSB extremely thermostable DNA-binding protein, cp/rxn copies per reaction.

Next, we optimized reaction parameters to further improve assay performance (Fig. 2a). To identify beneficial effects of modifications more robustly, we tested 16 replicates at 25 copies/reaction and measured improvements in detection rate. A progressive reduction of reaction temperature from 65 to 60 °C had the greatest effect. Other additively beneficial modifications were increasing Mg^2+^ concentration to 9 mM and the inclusion of 0.8 M betaine, 0.05% Triton X-100 and ET SSB, the latter we decided not to include in our master mix because of its high cost per reaction. With the revamped master mix, we ran a colorimetric LoD experiment for the two most promising sets and found the number of amplified replicates increased substantially for both (Fig. 2b). From these two sets, we picked E2L for further development because of its consistently lower time to reaction.

Finally, while screening E gene primer set variants, we noticed that related sets with lower F2/B2 *T*_*m*_ tended to exhibit faster amplification and sometimes higher sensitivities. We synthesized variants of the E2L set with different predicted *T*_*m*_ (~52, 55, and 58 °C) for their B2 and F2 regions and found that lower *T*_*m*_ in both regions led to faster amplification (Fig. 2c). Next, we tried to increase the concentration of auxiliary primers and found that increasing loop primers (from 400 to 600 nM; group 1.5 × LF/LB), but not outer primers (from 200 to 400 nM; group 2 × F3/B3), further improved reaction speed without impacting sensitivity or non-specific amplification (Fig. 2d). All these modifications resulted in a final E gene primer set (E2.2 L) with an LoD of ~25 copies/reaction of synthetic viral RNA and time to reaction around 20 min.

Multiplexing LAMP primer sets has been successfully demonstrated by multiple groups^27,41,64–67^ and leads to increased target detection sensitivity and consistency, provided strong primer interactions are absent. To test this approach, we picked a LAMP set targeting N gene designed by Mammoth Bioscience^28^ called DETECTR-N2 (or N2 here for simplicity) that has exceptional sensitivity (in low 10 s of copies/reaction)^28,41^ and introduced LNAs into its F3/B3 primers to further improve the set. As can be seen in Supplementary Fig. 2a, the LNA-modified version named N2L showed both improved detection rate and reaction speed, which was synergistic with the introduction of another modification, a linker in FIP. The final set (N2.2L) showed remarkable sensitivity, consistently detecting as few as 25 copies/reaction in ~20 min when evaluated by fluorescence. Moreover, the addition of the linker disrupted and mitigated non-specific primer interactions that resulted in late non-specific amplification observed in the no template controls of the original and LNA-modified primer sets. By multiplexing this N gene set (N2.2 L) together with our E gene set (E2.2 L), the combined assay detected 5 out of 8 replicates at 10 copies/reaction, easily outperforming both singleplex assays in terms of sensitivity and speed (Supplementary Fig. 2b).

### Temperature-dependent effects of guanidinium salts on reaction speed and non-specific amplification

While our E + N LAMP set mix was sensitive, the average time to reaction was almost 40 min (some replicates amplified as late as 60 min), which is worsened by colorimetric detection because it lags TTR by 5+ min. To accelerate the reaction, we added guanidinium chloride (GuCl) to the master mix, an additive that previous reports^55,64,68^ have shown can considerably enhance LAMP amplification rate. While addition of GuCl into our master mix improved reaction speed, the effect was minimal, limited to low concentrations (20 nM), and produced considerable non-specific amplification (Supplementary Fig. 2c). Since the melting temperature of products rapidly increased with increasing concentrations of guanidinium salts in the master mix (Supplementary Fig. 2d), we explored the possibility that the combination of low reaction temperature and guanidinium-induced increase in effective primer *T*_*m*_ hinders LAMP amplification. To follow up, we repeated this experiment at higher temperatures (62.5 and 65 °C) and compared GuCl with guanidinium isothiocyanate (GuSCN). Surprisingly, these higher reaction temperatures, which we originally identified as unfavorable, unmasked the full effect of guanidinium salts on amplification speed (Supplementary Fig. 2e, f), leading us to select 40 mM GuSCN as the different anion both accelerated the reaction and suppressed undesirable non-specific amplification (Supplementary Fig. 2e-f).

### An ultrasensitive triplexed assay detects down to 10 copies/reaction

**Fig. 3:**
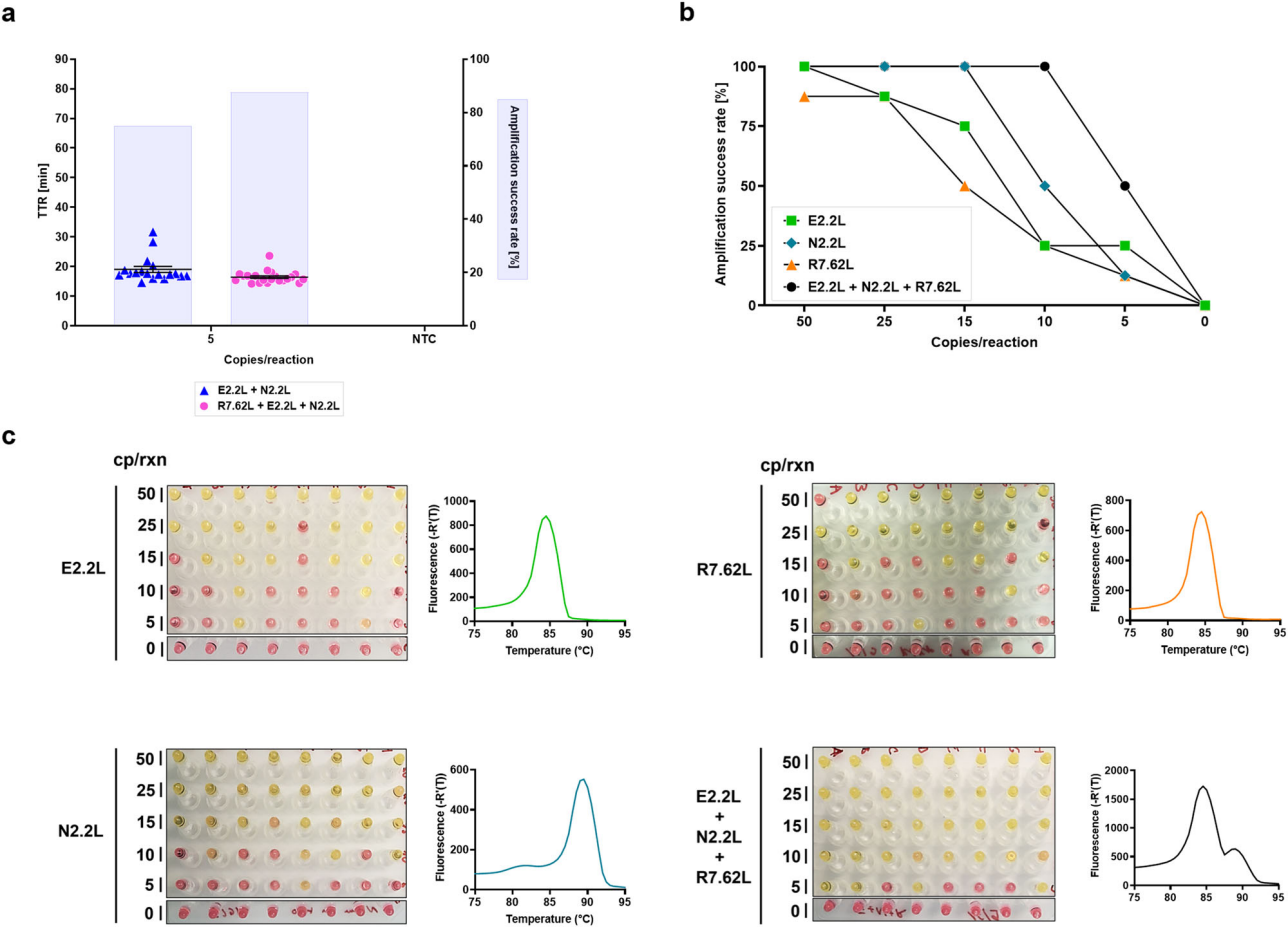
Primer set triplexing with RdRP gene detection and its impact on sensitivity. **a** Comparison of duplexed (E2.2 L + N2.2 L) and triplexed (E2.2 L + N2.2L + R7.62 L) primer sets at sub-limit of detection amounts of RNA template input. *n* = 24 technical replicates per group were used. **b**, **c** Analytical limit of detection experiment with triplexed combination of E2.2 L + N2.2 L + R7.62 L primer sets compared to individual primer sets. Experiments were run for 45 min and were evaluated by colorimetric detection only. Open symbols represent non-specific products, whereas closed symbols are specific as determined by melt curve analysis of amplification products. The highest time to reaction value on y-axis signifies the total duration of the reaction. Amplification success rate shows the percentage of samples that amplified over the course of the reaction. For NTC reactions, PCR-grade water was used as the input. All experiments were performed using the “NEB WarmStart 1.2” reaction mix. Error bars represent standard error of the mean. TTR time to reaction, NTC no-template control, cp/rxn copies per reaction.

Our internal target was to achieve an analytical LoD (95% detection rate) for synthetic RNA targets of ≤15 copies/reaction. Our desired level of sensitivity would allow the assay to completely bypass any type of laborious RNA extraction/concentration and still be able to detect low viral titers^69,70^ (<50 viral RNA copies/µl sample) in clinical specimens even with modest sample input volumes (~10% of reaction volume). In search of additional sensitivity, some groups have attempted to implement crude purification and concentration protocols, for example by using silica mesh/gel^55^, carboxylated nucleic acid capture beads, or dramatically increasing the reaction volume along with reagent usage and cost^71^. We tested some of these approaches but abandoned them as the extra steps resulted in a higher likelihood of user errors and longer assay times, thus negating a large advantage of LAMP over PCR. Instead, we designed and screened another LAMP set to multiplex with our E2.2 L and N2.2 L sets. We designed 8 sets (+ variants) targeting the RdRP gene and after multiple screening experiments we ended up with a compatible set we named R7.62 L. Triplexed E2.2 L + N2.2 L + R7.62 L reactions, compared with duplexed E2.2 L + N2.2 L reactions, demonstrated faster amplification rates, less variance in TTR, and detected 80+% of replicates at 5 copies/reaction (Fig. 3a). As a final test, we performed analytical LoD experiments (reaction time capped at 45 min) with this triplex combination, which unambiguously demonstrated greater sensitivity than any individual primer set (Fig. 3b, c).

### pH-independent colorimetric detection of LAMP amplification

With an ultrasensitive triplexed LAMP primer mix, our next step was to test our combination of primers and the modified NEB WarmStart master mix on clinical sample specimens. During the COVID-19 pandemic, many groups have reported that clinical specimens present challenges for classical pH-dependent colorimetric detection systems^55–59^. Saliva and/or gargle specimens tend to be acidic and may change the color of WarmStart or other Phenol red-based master mixes to orange/yellow even before amplification, while viral transport media may delay or prevent color change altogether, depending on their buffering pH and strength. Indeed, like others, we observed sporadic WarmStart discoloration even when using different lots of nuclease-free water. Unfortunately, this behavior was expected as WarmStart is essentially unbuffered and cannot tolerate samples with even low buffering strength. A frequently used alternative is HNB, which acts as a Mg^2+^ complexometric dye. Pyrophosphate produced during amplification sequesters free Mg^2+^ driving the somewhat ambiguous color change (from dark blue to lighter blue) of this metallochromic indicator as it loses coordinated Mg^2+^ ions^40^. While this system allows buffering, its sensitivity depends on master mix Mg^2+^ concentration, which in our case was high (9 mM).

**Fig. 4:**
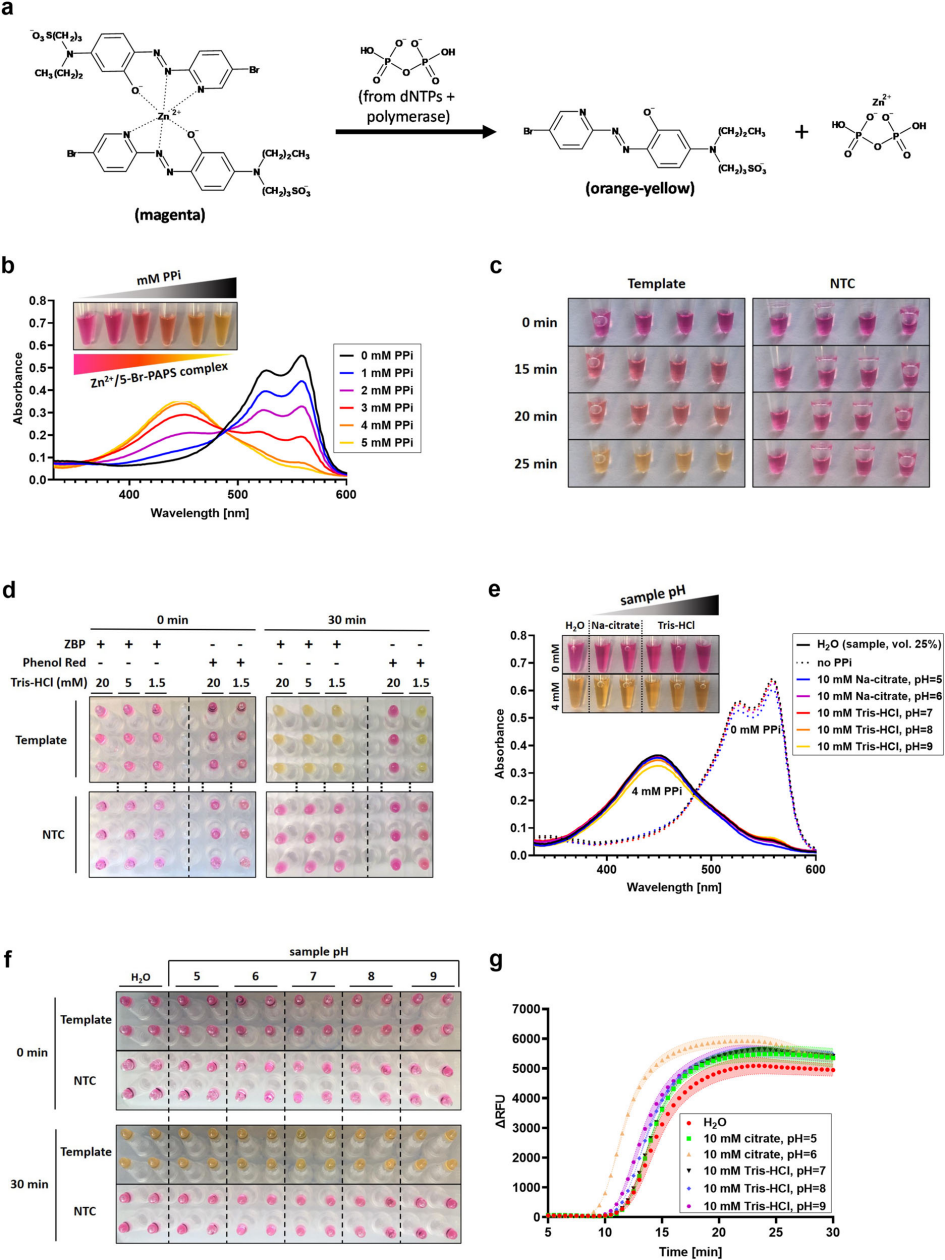
Development of a colorimetric detection method based on Zn^2+^/5-Br-PAPS. **a** Proposed mechanism behind the color change of the Zn^2+^/5-Br-PAPS detection reagent during LAMP amplification. The reagent changes color from magenta to orange-yellow that is dependent on the concentration of pyrophosphate, which is produced when DNA polymerase incorporates a nucleotide into a nucleic acid strand. **b** Absorption spectrum of the reaction mix including ZBP reagent changes progressively by increasing the pyrophosphate concentration. Specifically, the 560 nm absorption peak decreases while the 448 nm peak increases. The photo inset demonstrates that these changes in absorption spectrum due to increasing pyrophosphate concentration correspond to an easily discernible color change from magenta to orange-yellow. **c** Time course of ZBP reagent color transition. A progressive change in color from magenta to orange-yellow was observed only in samples where template was included and amplification occurred. **d** Comparison of tolerance to reaction mix buffering between a Phenol Red (“PR development” reaction mix) detection system and the ZBP detection system. The ZBP reagent changed color upon successful LAMP amplification even in the presence of up to 20 mM Tris-HCl pH = 7.9. All reactions were performed in triplicate. **e** Color transition of the ZBP reagent is retained even with the addition of different pH buffers. Samples with no PPi represent negative/pre-amplification samples while those with 4 mM PPi mimic samples where amplification did take place. Both spectrophotometric and visual data show that the behavior of the ZBP reagent is unchanged by the addition of 25% (v/v) of 10 mM buffers with various pH values. **f**, **g** Performance of the ZBP reagent in reactions with input samples buffered to different pH levels. A 30-min LAMP reaction was performed with a primer set targeting human RPP30 gDNA and monitored real-time. Samples with template prepared in different pH buffers did not negatively impact the resulting color change **f** or amplification **g**. *n* = 4 technical replicates per group were used. Dashed lines in **g** represent standard error of the mean along the individual curves. Unless specified otherwise, all experiments were performed using the “ZBP development” reaction mix. TTR time to reaction, NTC no-template control, RFU relative fluorescence unit, PPi sodium pyrophosphate, ZBP Zn^2+^/5-Br-PAPS complex.

Instead of using HNB or related compounds, we designed a colorimetric metallochromic dye-based detection system. We introduced a low micromolar concentration of a new ion into the reaction mix, Zn^2+^, along with a highly specific Zn^2+^ metallochromic indicator, 5-Br-PAPS. Zn^2+^ forms extremely stable and insoluble complexes with pyrophosphate, a feature exploited in the gravimetric detection of zinc^72^, while 5-Br-PAPS has been previously used in assays for measuring free Zn^2+^ by forming a deeply colored Zn^2+^ complex (hereafter referred to as ZBP)^73^. The proposed reaction of ZBP and pyrophosphate can be seen in Fig. 4a. This complex shares the main benefit of the HNB-Mg^2+^ detector, high tolerance to buffering (our ZBP master mix has 20 mM Tris-HCl by default), but additionally has two distinct advantages. First, master mixes utilizing ZBP can have their Mg^2+^ concentration optimized with little if any impact on colorimetric detection. Second and uniquely important for the development of a potential POCT not utilizing fluorescent detection, the color change ZBP undergoes during Zn^2+^ sequestration is visually striking: from magenta to orange-yellow. This contrast even allows unambiguously identifying positive samples with unfinished amplification (Fig. 4b, c).

As shown in Fig. 4d, despite the buffered master mix, progressively higher concentrations of pyrophosphate in the reaction cause a vibrant color change that can be observed throughout LAMP amplification (Fig. 4b, c). ZBP color change resembles the color transition of Phenol Red but in contrast it occurs at both low (1.5 mM) and high (20 mM) levels of master mix buffering (Fig. 4d). Additionally, these spectral characteristics (Fig. 4e) and associated colorimetric change (Fig. 4f) are unaffected by sample pH even with reasonably high buffering strength and input volume (10 mM, 25% of total reaction volume). When ZBP master mix was tested in a LAMP reaction, varying the pH of the input sample never impaired amplification as demonstrated by the progression and end-result (Fig. 4g). Together, these properties make ZBP ideal for real-time or end-point colorimetric monitoring of LAMP amplification of samples with either varying composition, pH substantially deviating from master mix pH, or strong buffering.

**Fig. 5:**
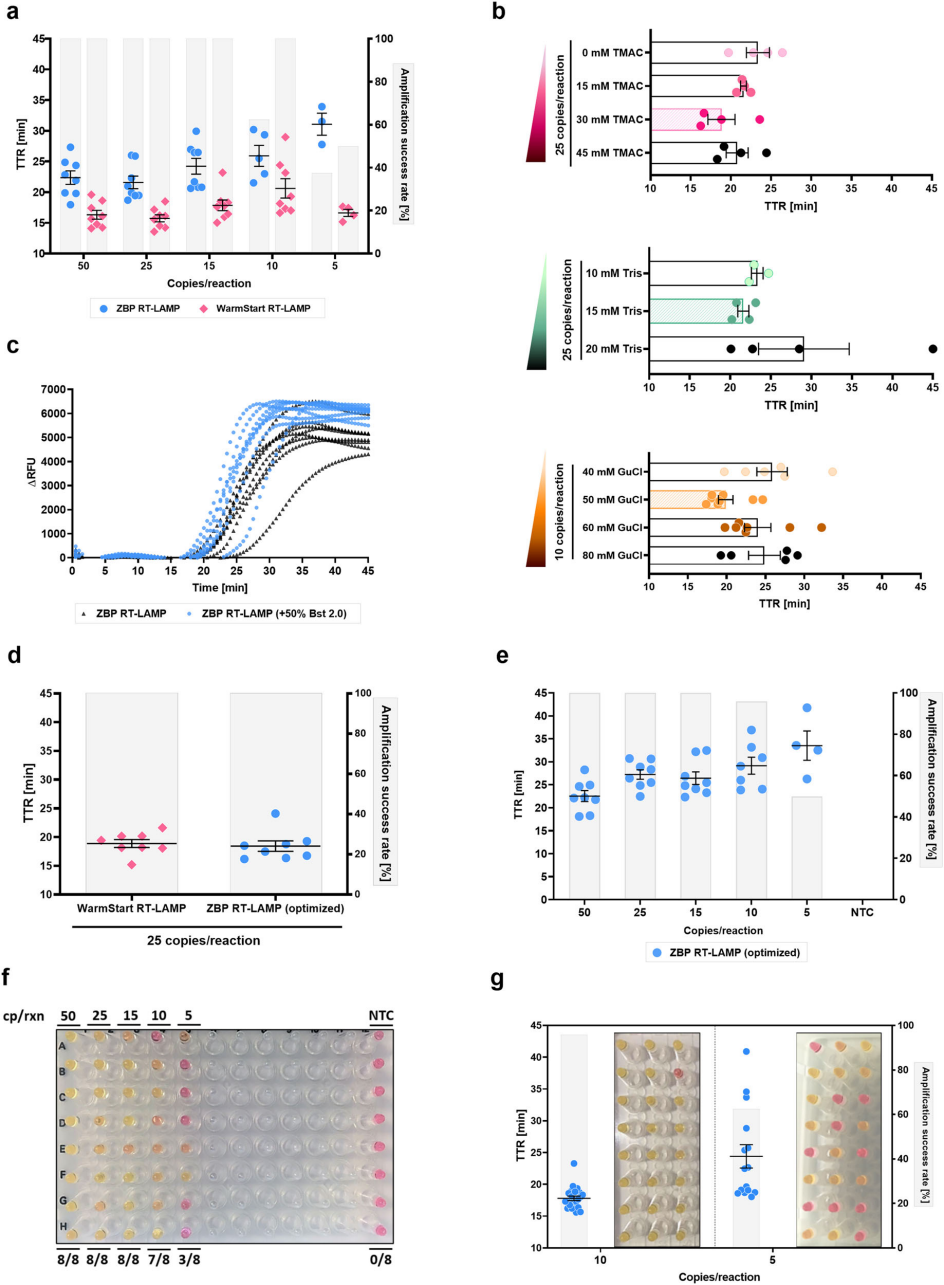
Optimization of RT-LAMP master mix using Zn^2+^/5-Br-PAPS reagent for colorimetric detection of SARS-CoV-2 with a triplexed LAMP reaction. **a** Detection limit of unoptimized “ZBP 1.0” reaction mix compared to the “NEB WarmStart 1.2” reaction mix. **b** Effects of modification of “ZBP 1.0” reaction mix constituents. KCl was substituted for TMAC and Tris/GuCl concentrations were optimized. The most optimal condition per experiment has its bar patterned. Unlike other experiments, *n* = 4 technical replicates per group were used for TMAC and Tris concentration testing. **c** Amplification plots showing the effects of an increased Bst 2.0 concentration (blue closed circles) versus standard Bst 2.0 concentration (black triangles) in ZBP RT-LAMP on amplification kinetics. **d** Comparison of optimized “ZBP 1.1” reaction mix with “NEB WarmStart 1.2” reaction mix. Limit of detection of optimized “ZBP 1.1” reaction mix shown as amplification success rate and TTR **e** and color change **f**. **g** Visual and fluorescent data for an extended 24-replicate limit of detection experiment with 10 and 5 copies of viral RNA per reaction. All samples positive by fluorescence changed color. Open symbols represent non-specific products, whereas closed symbols are specific as determined by melt curve analysis of amplification products. The highest time to reaction value on y-axis **a**, **d**, **e**, **g** or x-axis **b**-**c** equals the total duration of the reaction. Amplification success rate shows the percentage of samples that amplified over the course of the reaction. For NTC reactions, PCR-grade water was used as the input. Error bars represent standard error of the mean. Unless explicitly stated otherwise, *n* = 8 technical replicates per group were used. TTR time to reaction, NTC no-template control, RFU relative fluorescence unit, GuCl guanidinium chloride, TMAC N,N,N,N-tetramethylammonium chloride, cp/rxn copies per reaction, ZBP Zn^2+^/5-Br-PAPS complex.

Next, we applied this colorimetric detection system (ZBP RT-LAMP) to our triplexed SARS-CoV-2 LAMP assay. While the reaction worked as expected, it demonstrated somewhat lower sensitivity (5/8 replicates at 10 copies/reaction) and amplification speed compared with the WarmStart version of the master mix (Fig. 5a). Slower amplification was the greater issue as a sizable proportion of low copy number reactions did not undergo full color transition. We reasoned this might be due to the inhibitory properties of Zn^2+^, which have been observed in PCR^74^, or because of the inclusion of Tris in the master mix. To rectify this, we tried a few modifications/optimizations (Fig. 5b). We partially replaced KCl with N,N,N,N-tetramethylammonium chloride (TMAC), a known PCR enhancer^75^, identifying 30 mM as its optimal concentration as well as reduced Tris concentration from 20 to 15 mM. Finally, we replaced GuSCN with GuCl, as the slower amplification speed removed the issue of sporadic earlier non-specific amplification, and reoptimized its concentration to 50 mM. Next, we found that increasing Bst 2.0 polymerase concentration not only reduced the time to threshold but also increased the amplification slope steepness, implying a quicker full color transition (Fig. 5c). In a head-to-head comparison, the optimized ZBP RT-LAMP assay demonstrated performance roughly equivalent to the original WarmStart assay (Fig. 5d). Retesting the LoD confirmed that the implemented changes improved sensitivity (Fig. 5e, f). To thoroughly characterize this final ZBP RT-LAMP assay, we performed an extended LoD experiment with 24 replicates at 10 and 5 copies/reaction (Fig. 5g), which showed remarkable detection rates at these low template amounts (95% and 62.5% respectively by colorimetric change).

### Adapting ZBP RT-LAMP to direct samples

**Fig. 6:**
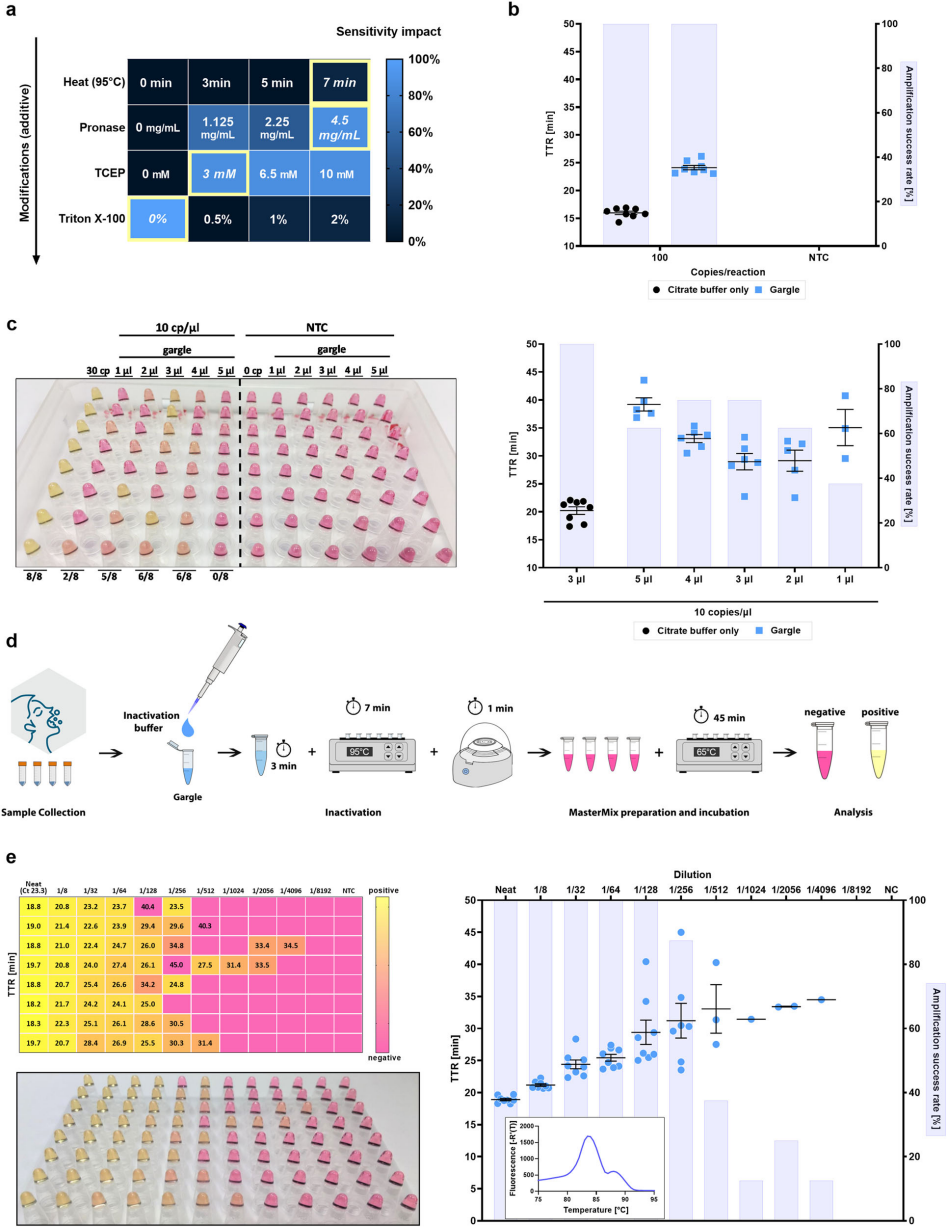
Modification of ZBP RT-LAMP assay for detection of SARS-CoV-2 from crude gargle samples. **a** Heat map depicting the optimization of heat protocol and components of 10 × inactivation buffer. The conditions highlighted in yellow depict the optimal parameter that was carried over to the next testing phase; therefore, modifications are additive going from top to bottom. **b** Comparison of amplification characterstics between reactions run with gargle versus 10 mM citrate buffer (pH = 6) as input. Both sample types first underwent the full inactivation procedure before being spiked with the same amount of RNA. **c** Colorimetric and fluorescent performance of RT-LAMP with different volumes of gargle spiked with 10 copies of viral RNA per µl of gargle compared to 30 copies of viral RNA in 10 mM citrate (pH = 6) used as reference. Identical gargle volume was used in NTC reactions. **d** Scheme demonstrating the workflow for LAMP based detection of SARS-CoV-2. Gargle collection is followed by the inactivation, heat-treatment, and short centrifugation to exclude proteineous precipitates. Inactivated samples are then added to prepared master mix, incubated for 45 min at 65 °C, and analyzed by visual confirmation by the naked eye. **e** Detection limit of Direct ZBP RT-LAMP assay using a mock clinical gargle sample. Depicted Ct of the neat sample that was determined by RT-qPCR and served as the reference point for serial fold-dilution. Exemplary melt curve of LAMP products is shown as an inset. The highest time to reaction value on y-axis equals the total duration of the reaction. Amplification success rate represents the percentage of samples that amplified over the course of the reaction. Gargle without viral RNA was used in NTC reactions. All experiments were performed using “ZBP 1.1 G” reaction mix. Error bars represent standard error of the mean. In all experiments, *n* = 8 technical replicates per group were used. NTC no template control, TTR time to reaction, TCEP Tris(2-carboxyethyl)phosphine, cp/µl copies per microliter.

Encouraged by reports of direct RT-LAMP assays that detect SARS-CoV-2 in clinical specimens with minimal sample processing^22,23,41,47,48,55,71,76–78^, we sought to develop a sample inactivation procedure compatible with our assay while preserving viral RNA and consequently assay sensitivity. As our primary clinical specimen type, we picked isotonic saline gargle because it has minimal variation in its composition (unlike viral transport media), does not have the consistency/homogeneity issues of saliva, and can be non-invasively self-collected by most people. For our base 10 × Inactivation buffer, we selected a sodium citrate buffer (100 mM, pH = 6), which doubles as a metal ion chelator, supplemented with carrier RNA (100 µg/ml). To reduce the risk of amplicon carry-over contamination, we modified the master mix to include Antarctic Uracil DNA Glycosylase and 20% dUTP instead of dTTP^79^. Besides testing a simple heating step to inactivate RNases, we also assessed the effect of Pronase (up to 4.5 mg/ml in 10 × Inactivation buffer), a proteolytic mixture from *Streptomyces griseus*, and the reducing agent TCEP (Fig. 6a). We chose Pronase instead of the much more commonly used proteinase K for its wider substrate specificity and lower stability towards heat inactivation. Heat by itself was insufficient and even 7 min of heating at 95 °C did not allow us to detect 30 RNA copies/reaction (3 µl input, 10 RNA copies/µl in input sample). However, inclusion of a 3-min Pronase incubation step before the 7-min heating dramatically improved assay performance. This was further synergistic with the inclusion of TCEP, a reducing agent. While this crude sample processing dramatically improved sensitivity, reactions with gargle as input versus equivalent reactions with citrate buffer only were noticeably slower even with high template amounts (Fig. 6b). Additionally, we also tried adding Triton X-100, a surfactant, into the inactivation buffer but unlike a previous report^80^ but in agreement with^65^, even small amounts completely abolished amplification (Fig. 6a), possibly by prematurely releasing RNA and/or enhancing RNase activity.

Some groups have reported that increasing the sample input volume up from 1 µl in direct LAMP does not appreciably affect reaction sensitivity^65,68^. For all experiments, we used 3 µl of inactivated gargle as input (15% total reaction volume) and wondered whether changing the input might help. However, we found that in direct ZBP RT-LAMP, 3 µl gargle input is optimal in terms of both speed and sensitivity (Fig. 6c).

After establishing our final gargle testing workflow (Fig. 6d), we tested the sensitivity with a mock clinical gargle sample by spiking negative gargle with live SARS-CoV-2 virus. We inactivated the sample according to the procedure described above and then performed a serial fold-dilution experiment (diluted with the same inactivated negative gargle) to empirically estimate the assay LoD on direct gargle samples (Fig. 6e). As a reference point, we also determined the SARS-CoV-2 E gene Ct value from the extracted RNA of the same mock sample with a certified RT-qPCR test used for routine testing. The mock sample Ct was 23.3, which based on a qPCR standard curve constructed from a quantified commercial standard, a concentration factor during RNA extraction, and sample input volume (5 µl) implies a concentration of ~2 × 10^6^ viral genome copies/ml. We managed to colorimetrically detect all replicates down to the implied Ct of 29.3 (~3.2 × 10^4^ viral genome copies/ml) and 6 replicates (75%) at 2 Ct values higher (~8 × 10^3^ viral genome copies/ml). Detection rates by fluorescence (Fig. 6e) were only marginally better, suggesting that colorimetric detection should be the method of choice due to its ease and undemanding equipment requirements. Melt curve analysis showed a product profile analogous to the RNA RT-LAMP variant of the assay (Fig. 6e), confirming that our triplexed SARS-CoV-2 LAMP primers display consistent behavior across different conditions – whether with synthetic RNA in water or when assaying live virus in a gargle background.

### Quadruplexing and reaction mix modifications further improve ZBP RT-LAMP – Vivid COVID-19 LAMP

**Fig. 7:**
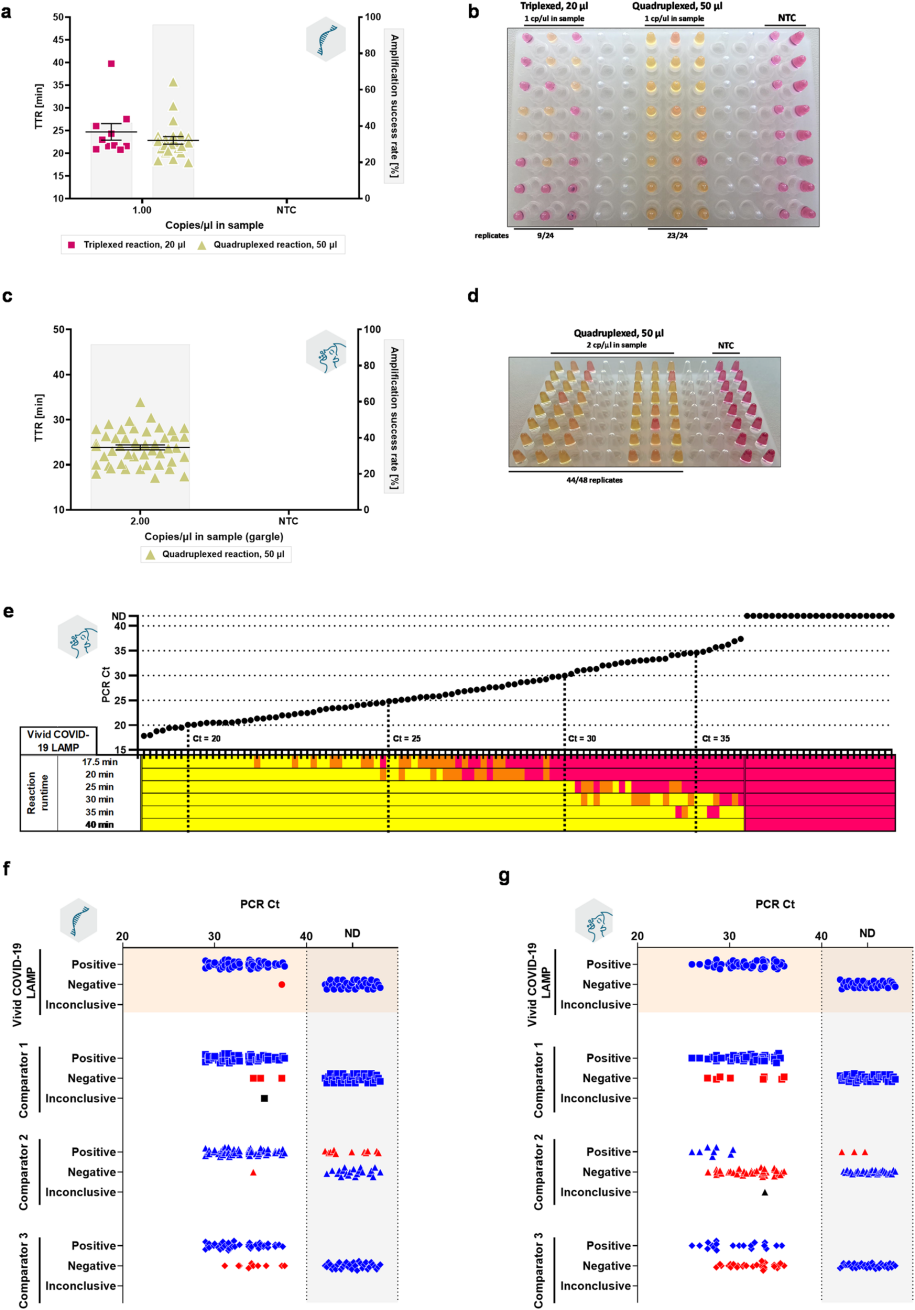
Quadruplexing and increasing reaction volume dramatically improves performance. **a**, **b** Comparison of extended LoD experiments for the original 20 µl triplexed reaction and 50 µl quadruplexed reaction at 1 synthetic RNA/µl input sample. The chosen template concentration corresponds to 3 copies/reaction in the original 20 µl reaction format and 8 copies/reaction in the 50 µl reaction format. *n* = 24 technical replicates were used for groups with SARS-CoV-2 RNA template while *n* = 8 technical replicates were used for NTC reactions instead. **c**, **d** Performance comparison of the original 20 µl triplexed reaction and 50 µl quadruplexed reaction with gargle specimens spiked with a given concentration of inactivated SARS-CoV-2 virions. *n* = 48 technical replicates were used for groups with SARS-CoV-2 RNA template while *n* = 16 technical replicates were used for NTC reactions instead. **e** Determination of optimal reaction runtime for the quadruplexed 50 µl version of the assay (Vivid COVID-19 LAMP) with 96 gargle specimens (1 technical replicate per 1 biological replicate) spiked with increasing concentrations of live SARS-CoV-2 virus (BA.5 variant) and 24 negative gargle specimens. Individual points denote Cts as determined by RT-qPCR and colored cells underneath represent observed colorimetric reaction results as per color chart developed for this assay (Supplementary Fig. 5) after a predetermined amount of time has elapsed (red – negative, orange – inconclusive, yellow – positive). Dotted lines represent arbitrarily chosen RT-qPCR threshold Ct values in 5-unit increments. Comparison of the quadruplexed 50 µl version of the assay (Vivid COVID-19 LAMP) against three other SARS-CoV-2 LAMP assays on either a panel of low-viral load extracted RNA **f** or direct gargle **g** samples. Samples (96 positive and 24 negative; 1 technical replicate per 1 biological replicate) were evaluated based on colorimetric detection. Data points colored in red mark false negative (Ct ≤40) or false positive (Ct = ND) samples. Data points colored in black denote samples with intermediate color change. ZBP 1.1 G and ZBP 2.0 reaction mixes were used for 20 and 50 µl reaction formats, respectively. Sample input volume was kept roughly proportional to the final reaction volume (3 µl in 20 µl and 8 µl in 50 µl). The highest time to reaction value on y-axis equals the total duration of the reaction. Experiments where RNA was used as input are marked with a single stranded RNA symbol, whereas experiments with gargle as input are marked with a gargling person symbol. Amplification success rate represents the percentage of samples that amplified over the course of the reaction. Error bars represent standard error of the mean. NTC no template reaction, ND not detected, TTR time to reaction, cp/µl copies per microliter, Comparator 1 Colorimetric ReadiLAMP™ Kit from Thermo Fisher Scientific, Comparator 2 SARS-CoV-2 Rapid Colorimetric LAMP kit from New England Biolabs, Comparator 3 SARS-CoV-2 LAMP assay adapted from ref. ^55^.

By increasing the reaction volume to 50 µl, we were able to increase the optimal sample input to 8 µl (16% of total reaction volume), which resulted in additional gains in sensitivity and speed (Supplementary Fig. 3a). Increasing the reaction volume ultimately requires additional master mix and enzymes that increase cost per reaction. To offset this cost increase, we modified enzyme ratios and managed to maintain the same sensitivity while reducing cost per reaction. Next, we introduced a 4^th^ primer set into the reaction (an LNA-modified version of the As1e set reported by^55^ and labeled As1.2 L in Supplementary Data 2) and compared this quadruplexed assay to the triplexed version using the same master mix composition and 50 µl reaction volume (Supplementary Fig. 3b). Quadruplexing yielded extra sensitivity both with RNA (Supplementary Fig. 3b–d) as well as direct gargle as input (Supplementary Fig. 3e, f) and also made the assay more robust towards emerging SARS-CoV-2 variants. Increasing the number of LAMP primer sets in a single reaction beyond three is thus a viable approach despite high primer interaction potential. This assay we named Vivid COVID-19 LAMP surpassed the original triplexed 20 µl version of the assay (Fig. 7a–d) and could detect spiked gargle samples in a direct format (i.e., no RNA extraction) with min viral loads (2 copies/µl sample or 16 copies/reaction in direct RT-LAMP, Fig. 7c, d) with shorter reaction times (40 min).

With this final assay, we thoroughly characterized the relationship between required amplification time and target sensitivity by colorimetric detection. We tested the assay on a panel of gargle specimens spiked with live virus (Ct range of 17.8-37.4) with varying reaction runtimes (Fig. 7e**)**. As expected, progressively shorter reaction runtimes precluded lower viral load samples from changing color (Fig. 7e, Supplementary Fig. 4) and a 40 min runtime was found to be necessary to completely amplify all samples, including ones with low viral loads; however, shorter runtimes could be utilized if lower assay sensitivity is warranted by the testing conditions such as testing of clinically symptomatic patients in hospitals, clinics, and doctor offices (25 min for medium load samples up to Ct 30 or 20 min for high load samples up to Ct ~25).

The performance of our final assay, Vivid COVID-19 LAMP, was subsequently compared to three state-of-the-art colorimetric SARS-CoV-2 LAMP assays, two commercial kits (Invitrogen™ Colorimetric ReadiLAMP™ Kit for SARS-CoV-2 by Thermo Fisher Scientific and SARS-CoV-2 Rapid Colorimetric LAMP Assay Kit by New England Biolabs, NEB) and the LAMP assay developed by Rabe & Cepko^55^, on a panel of extracted RNA and direct gargle samples with 48 low viral load positive samples and 32 negative samples (Fig. 7f, g, Supplementary Fig. 5). With extracted RNA our developed assay demonstrated equivalent sensitivity to the NEB assay detecting all but a single sample in the tested panel (Ct range 29.0–37.6) with Thermo Fisher’s ReadiLAMP™ being a close second (Fig. 7f). However, the NEB assay showed a high degree of non-specific amplification with negative RNA samples. We consistently observed this behavior during initial testing of the kit with it being worse with less controlled sources of heating (e.g., dry block heater without a heated lid instead of RT-qPCR system), suggesting that evaporation may result in non-specific amplification. Indeed, such behavior of this kit has been reported previously and the non-specific amplification was mitigated with a mineral oil overlay^81^. With direct gargle samples (Ct range 25.9–35.9) as input, the Vivid COVID-19 assay performed the best by a substantial margin as it detected all tested samples with unambiguous color change (Fig. 7g, Supplementary Fig. 5) followed by Thermo Fisher’s ReadiLAMP™. Both NEB and Rabe & Cepko assays had much lower sensitivities with this sample type with NEB additionally showing some non-specific amplification. However, one should note that the NEB assay has not been optimized for this sample type and it is likely better results could be obtained with proper modifications. Summarily, the Vivid COVID-19 LAMP assay outperformed with direct gargle inputs, while having exquisite sensitivity with extracted RNA on par or better than that of the other tested assays (Supplementary Data 3).

### Clinical performance of triplexed and quadruplexed SARS-CoV-2 assays

**Fig. 8:**
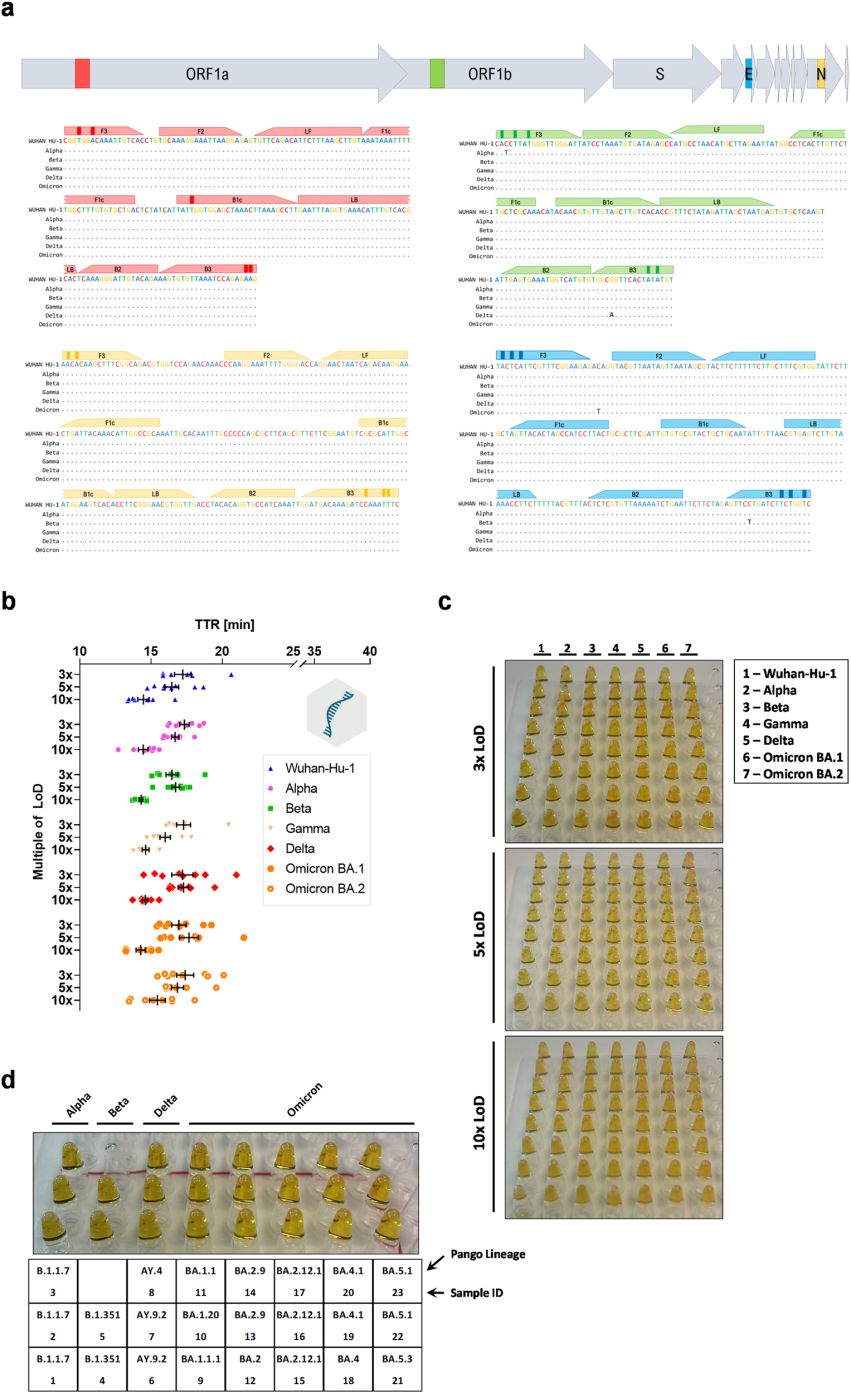
LAMP primers detect all major SARS-CoV-2 variants of concern. **a** Schematic illustrating the SARS-CoV-2 genome and regions targeted by the LAMP primer sets used in the SARS-CoV-2 ZBP RT-LAMP and Vivid COVID-19 LAMP assays. Priming regions are aligned against consensus sequences of Alpha, Beta, Gamma, Delta, and Omicron variants of concern. LAMP primer sets are color-coded: red – As1.2 L, green – R7.62 L, blue – E2.2 L, yellow – N2.2 L. Dark-colored bars in primers represent LNA-modified nucleotides. **b**, **c** Demonstration of Vivid COVID-19 LAMP reactivity against variant-specific synthetic RNA controls at indicated multiples of LoD. **d** Demonstration of Vivid COVID-19 LAMP reactivity against variant-specific isolated RNA patient samples with sequencing-confirmed lineage status. Sample ID represents table entry in Supplementary Data 5 with additional sample information. 1 technical replicate per 1 biological replicate was tested. Error bars represent standard error of the mean. Unless explicitly stated otherwise, *n* = 8 technical replicates per group were used. LoD limit of detection, TTR time to reaction, ORF open reading frame, S spike, E envelope, N nucleocapsid.

Before clinical validation we performed cross-reactivity and inclusivity analyses. In silico cross-reactivity analysis revealed only the original SARS-CoV to be of potential concern (Supplementary Data 4); however, in wet-lab testing, all viral pathogens (including SARS-CoV) gave negative results (Supplementary Fig. 6a). During development, several variants of concern (VOC) emerged, including the latest Omicron variant, underscoring the essential nature of variant inclusivity analysis. Sequence alignments of primers used in our SARS-CoV-2 assays against generated VOC consensus sequences show excellent coverage with only rare mismatches isolated to a singular primer per VOC at most (Fig. 8a). Detection of the major VOCs by the Vivid COVID-19 LAMP assay was also confirmed experimentally on a panel of variant-specific synthetic controls at 10 ×, 5 × and 3 × of analytical LoD (Fig. 8b-c) as well as on low viral load patient samples with sequencing-confirmed lineage status (Supplementary Data 5), including the recently identified BA.2.12.1, BA.4, and BA.5 Omicron variants (Fig. 8d).

**Fig. 9:**
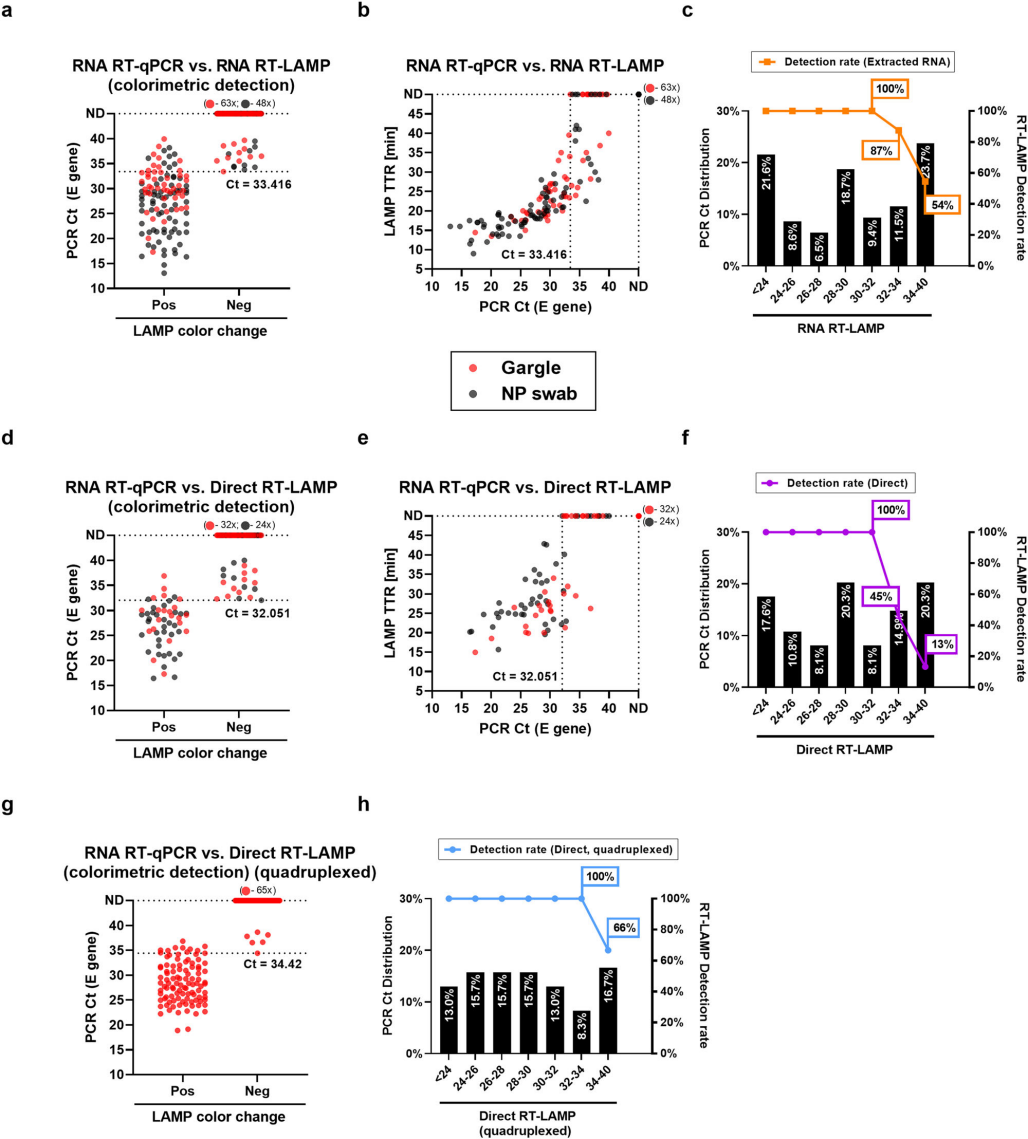
Clinical performance evaluation of SARS-CoV-2 ZBP RT-LAMP and Vivid COVID-19 LAMP. Evaluation of ZBP RT-LAMP using both **a** colorimetric and **b** fluorescent detection of extracted RNA from either clinical patient nasopharyngeal swab or gargle samples. **c** Detection rates of RT-LAMP relative to the distribution of RT-qPCR Ct values from samples analyzed in **a** and **b**. Evaluation of direct ZBP RT-LAMP using both **d** colorimetric and **e** fluorescent detection of RNA, without prior RNA extraction, from either clinical patient nasopharyngeal swab or gargle samples. **f** Detection rates of direct ZBP RT-LAMP relative to the distribution of RT-qPCR Ct values from samples analyzed in **d** and **e**. **g** Evaluation of Vivid COVID-19 LAMP using colorimetric detection of RNA, without prior RNA extraction, from clinical patient gargle samples. **h** Detection rates of Vivid COVID-19 LAMP relative to the distribution of RT-qPCR Ct values from samples analyzed in **g**. The highest time to reaction value on y-axis is equal to the total duration of the reaction. ND stands for samples which did not amplify over the course of the reaction. All reactions for ZBP RT-LAMP were performed using “ZBP 1.1 G” reaction mix while reactions for Vivid COVID-19 LAMP were conducted using “ZBP 2.0” reaction mix. Dotted lines outside of ones extending from ND ticks show the threshold for 100% detection by the tested LAMP assays. For all patient samples 1 technical replicate per 1 biological replicate was tested. Ct – PCR cycle threshold; TTR – time to reaction; NP - nasopharyngeal.

Lastly, we performed two separate clinical validations in a prospective setting: one for the triplexed lower volume version of the assay (direct ZBP RT-LAMP) with paired NP swab and gargle specimens from a total of 72 patients plus an additional 53 patients from whom only sample-extracted RNA was available; and a second validation for the quadruplexed higher volume version (Vivid COVID-19 LAMP) with 173 gargle specimens (Fig. 9a–h). Results from our LAMP assays were compared against an RT-qPCR test used for routine screening^82^ (distribution of determined Ct values can be seen in Fig. 9c, f, h). For colorimetric detection a color chart was used to classify final reaction color (Supplementary Fig. 6b).

ZBP RT-LAMP, both RNA (Fig. 9a–c) and direct (Fig. 9d–f) versions, showcased good results. Both colorimetric (Fig. 9a, d) and fluorescent detection (Fig. 9b, e) had significant agreement with the reference RT-qPCR method and in general there was a correlation between RT-qPCR Ct values and LAMP TTR. No false positive results were observed (NPA = 100%). With colorimetric detection, PPA in the whole sample set was 85.6% for RNA and 73.3% for direct versions, respectively. In both cases, applying a cut-off value of Ct <32 increased the PPA to 100%. Fluorescent detection rate was almost identical as few samples positive by fluorescence failed to change color (Supplementary Fig. 6c, d).

Vivid COVID-19 LAMP performed excellently with direct gargle samples with an overall NPA of 100%, while PPA by colorimetric detection was 94.44%. With a cut-off value of Ct <34 the PPA was 100% (Fig. 9g). Notably, our clinical validation had a high proportion of medium-to-low viral load samples as >50% samples had Ct values of ≥28 (Fig. 9h). The results affirm that Vivid COVID-19 LAMP has excellent overall agreement with RT-qPCR up to Ct 34 even when omitting an RNA extraction/concentration step. Details and subgroup analyses for both ZBP RT-LAMP and Vivid COVID-19 LAMP can be found in Supplementary Data 6.

### Adapting Vivid COVID-19 LAMP for field use

To demonstrate the potential of Vivid COVID-19 LAMP in real world POCT scenarios, we designed and created a semi-automated, mobile LAMP testing laboratory for medium- to high-throughput field testing (Supplementary Fig. 7). The laboratory is fully contained in a commercial van divided into two physically separated sections for sample inactivation and for LAMP amplification, thus maintaining a workflow that minimizes sample contamination from prior amplicons. The streamlined process (Supplementary Video 1) involves first pairing incoming samples with tubes pre-filled with our inactivation buffer both of which are linked by machine-readable barcodes. Then samples are inactivated, placed into barcoded 96-well racks with open bottoms, and transferred to the area of the van designated for LAMP amplification. Filled racks are scanned in the LAMP section of the van and a semi-automated 96-well pipettor is used to transfer inactivated sample supernatants into pre-filled LAMP plates followed by amplification. To identify poorly collected gargle specimens, a control assay targeting the RPP30 subunit of human RNase P is used to detect the presence of human genomic material and is performed in parallel with the SARS-CoV-2 LAMP assay (see Supplementary Fig. 6e for combined results interpretation). The outlined procedure allows anonymized and automated sample ID and position tracking, all tied together by a central database.

**Fig. 10:**
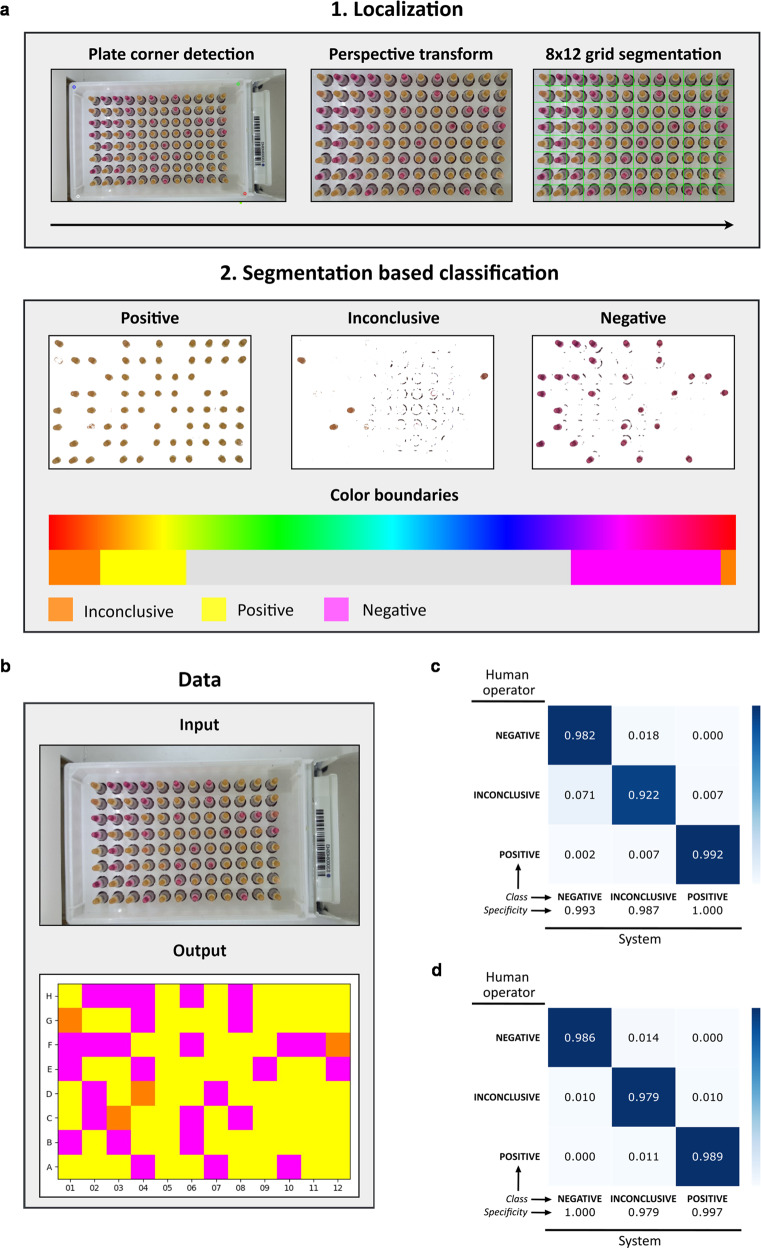
Machine-guided reaction color classification system. **a** Schematic representation of the approach utilized by the machine-guided determination of reaction color. The input image is first analyzed to find landmarks (four corners), then proceeds to perspective transform to normalize the image by cropping and rotating, and finally is segmented to obtain an 8 × 12 grid corresponding to 96 possible plate wells. Individual grid cells have a class assigned using an algorithm based on pixel hue determination with color saturation filtering. A color hue scale with depicted ranges corresponding to individual classes is shown below. **b** Example of input photo transformation into a final 8 × 12 grid with individual wells ascribed classes. Classes are represented by the fill color of a given cell representing an individual reaction. Confusion matrices (3 × 3) constructed from testing data for SARS-CoV-2 **c** and RNase P reactions **d**, respectively. The intensity of shaded squares and inscribed values represent the frequency of a given pair of human-system classifications out of all reactions in its row. Squares in the diagonal direction from upper left to lower right represent sensitivity of the developed system for a given class of results when compared to the judgment of a human operator. Specificity of detection for the same classes is shown underneath the main matrix.

Finished plates are then scanned in a 3D-printed light box with a tablet running custom-designed software for sample-level colorimetric LAMP analysis. The software was designed to reduce the hands-on time to classify final reaction colors, mitigate subjective biases in color interpretation, and permit high-throughput analysis (Fig. 10). Our approach is based on traditional computer vision techniques combined with neural network landmark localization. The system receives a photo of the plate as input and localizes the four corners to crop and align the plate (perspective transform) in the image to get sample ID and position. Next, the individual samples are segmented and classified based on color saturation in conjunction with a hue channel classification range (Fig. 10a). The system then transforms the input photo into an output matrix (8 ×12 resolution) with mapped positions of all samples with classification results (Fig. 10b).

### The performance of machine-guided plate analysis system and real-world field-testing results of Vivid COVID-19 LAMP

Using our test dataset obtained during National Public COVID-19 testing in Slovakia, the system had a sensitivity for positive/negative/inconclusive colors of 99.2%/98.2%/92.2%, respectively, and an overall sensitivity of 98.4% when judged against a human operator (i.e., only 70/4398 results were overridden by a human operator; Fig. 10c). Similar results were obtained for the RNase P reaction where the reaction volume and sample input is halved, and color intensity is somewhat different (Fig. 10d).

Overall, only 3 cases of false negative color classifications were detected, arising from a rare technical issue – insoluble material got into the reaction, sedimented, and sequestered the Zn^2+^/5-Br-PAPS complex, thus yielding a magenta-colored pellet. Since the photo was obtained from the bottom of the well plate, the system classified these reactions as negative, but human operators could effortlessly spot the bulk of the reaction volume being yellow. Importantly, after human intervention, these cases did not lead to a false negative result in practice.

The lowest sensitivity was observed for inconclusive samples mainly because our system flagged a portion of negative samples as inconclusive. This issue results from uneven lightning conditions between wells, which is exacerbated if the plate is closer to the camera than expected, resulting in certain wells receiving more illumination than others. On the other hand, edge wells can appear darker resulting in the reverse error. A possible solution is to redesign the light box to ensure more even illumination between wells or modifying our software to utilize deep learning techniques to increase its robustness under these conditions.

To demonstrate the utility of this internally developed version of Vivid COVID-19 LAMP for field testing, we successfully utilized it in a variety of real-world testing situations (details described in Supplementary Data 8), including the Globsec conference (June 2021), Pohoda on the Ground music festival (July 2021), Canoe Slalom World Championships (September 2021), Winter Olympics Men’s Hockey Qualification round (September 2021), Handball World Championships (January 2022), routine mobile testing during the Omicron wave in Slovakia (January-March 2022) as well as testing of Ukrainian refugees crossing the Slovak border (February-March 2022), employees of various private companies, and schoolchildren at a private school. Altogether, this involved conducting 8,733 LAMP tests, which, to our knowledge, importantly resulted in no false positives (verified by RT-qPCR in most cases) or false negatives.

## Discussion

LAMP is a sensitive and specific NAAT that provides a fast and affordable alternative to PCR and RATs for detecting infectious agents. The entire LAMP protocol can be performed isothermally^11^ and with colorimetric detection^45,83^, obviating the need for expensive laboratory equipment. Due to the high resistance of polymerases used in LAMP, the method can be paired with minimally processed patient samples even those rich in traditional PCR inhibitors. This combination is especially intriguing with respect to POCT and has been explored by multiple groups during the current COVID-19 pandemic^22,41,42,65,68,71,84–86^; however, its practical implementation has been marred by technical issues such as lower sensitivity with crude samples and lack of robust colorimetric detection. In this work, we introduce approaches to combat these difficulties including 1) using strategic placement of LNA-modified nucleotides in specific LAMP primers to increase sensitivity and speed; 2) optimizing an ultrasensitive quadruplexed combination of SARS-CoV-2-specific LAMP primer sets, a challenging feat in LAMP given the propensity for nonspecific amplification when combining multiple primer sets. Indeed, to our knowledge, the Harmony COVID-19 assay is the only other LAMP test that has successfully multiplexed four LAMP primer sets^66^; 3) developing a colorimetric detection system that does not rely on Mg^2+^, yields a perceptible, striking color transition, and is impervious to changes in pH, thus making it ideal for challenging direct sample types; 4) developing reagents and an inactivation procedure compatible with the Zn^2+^-based colorimetric detection system for assaying direct samples such as self-collected gargles; 5) and utilizing artificial intelligence and computer vision techniques to develop a mobile application that can be used to objectively classify and report colorimetric results. All these innovations culminate in Vivid COVID-19 LAMP, an ultrasensitive and specific assay compatible with both extracted RNA as well as minimally processed direct samples both in lab and field settings. We demonstrate the real-world utility of this assay by designing a mobile vehicle containing a laboratory and testing in a variety of POCT scenarios, including sporting events, schools, conferences, private companies, and country-wide testing.

Although multiplexing LAMP primer sets has been demonstrated before^27,41,64–67^, our efforts to successfully combine four LAMP primer sets into a single assay is a notable endeavor that only one other assay has achieved^66^. Given the number of individual primers, their high concentrations, and the properties of strand-displacing polymerases, LAMP reactions have the propensity to yield non-specific amplification products. To mitigate the influence of non-specific amplification, we incorporated several quality control measures including numerous replicates, monitoring of melt curve analysis to identify non-specific products, and sufficient negative controls. Even though we incorporated robust quality measures, it is important to highlight that our pH-independent ZBP colorimetric detection system is a sequence-independent detection system; that is, it cannot distinguish what amplified sequence is contributing to the color change. In contrast, sequence-dependent assays, such as probe/reporter-based assays allow the user to identify the gene targets that are being amplified, albeit with the necessary infrastructure required to detect several fluorescent signals. This distinction has recently been described in an excellent review paper^9^, where the authors highlight the limitations and benefits of both types of multiplexed assays and suggest that sequence-independent assays are perhaps more aptly described as “combiplex” assays. Nevertheless, colorimetric detection assays, especially those that do not rely on changes in pH, can provide high specificity and sensitivity, while also being more amenable to POCT conditions.

Compared with RT-qPCR, LAMP primer set design is more complex (6 or 8 priming regions), poorly characterized, and only a single free online tool (PrimerExplorer) based on the original LAMP patents and publications^11,12^ is available. This dramatically complicates the identification of sets sensitive enough for direct sample assays. It is possible the universally accepted primer recommendations are suboptimal especially with targets containing unideal GC content such is the case with SARS-CoV-2. Primer binding characteristics can be heavily influenced by the incorporation of modified/non-standard bases^87^ and this behavior is further influenced by the target template type^88^. A key consideration with LAMP is that due to the isothermal, continuous nature of the method, the steady-state concentration of a primer-template duplex should be more sensitive to the properties of the primers than in PCR. With these ideas in mind, we tested the judicious incorporation of LNAs near the 5’-end (preferring As/Ts over Gs over Cs to prevent unnaturally strong base pairing that promotes mis-priming and primer-dimer formation) of individual LAMP primers as this modification improves primer sensitivity and specificity in RT-qPCR applications^62,89,90^. While LNAs have been used in the context of LAMP before the COVID-19 pandemic, this was either to improve mismatch tolerance^91^ or implement allelic discrimination^92^, suggesting their use in LAMP to increase template binding strength remains relatively unexplored.

Intriguingly, only LNA-modified outer primers (F3/B3) showed consistent improvements in sensitivity/reaction speed (Fig. 1) while modifications in other primers were detrimental or neutral to performance (Supplementary Fig. 1). This suggests that the PrimerExplorer recommended primer *T*_*m*_ for F3/B3 regions with AT(U) rich templates are suboptimal as our LNA-modified primers had on average a predicted *T*_*m*_ that was 3–5 °C higher. A likely explanation is that the enhanced hybridization properties of LNA-modified F3/B3 primers accelerate strand displacement and consequently formation of dumbbell-like cDNA structures that start LAMP amplification, an effect that could be crucial with low-copy template reactions. In RT-LAMP, the B3 primer also initially interacts with an RNA template and as LNA/DNA hybrids preorganize the backbone into A-type-like conformation typical for RNA/RNA duplexes this could bestow further binding advantages. Consistent with our findings, Ludwig et al.^93^ report in their LAMP-Seq assay that LNA-modifications placed at the 5’ end and middle of F3/B3 primers increase assay sensitivity.

Loop primers were by far the worst recipients of LNA modifications displaying both reduced sensitivity and impeded reaction speed. LNA modifications of the B2/F2 regions of the BIP/FIP primers, which are key in recognizing the template, in general showed poorer performance to unmodified primers, in contrast to what one would predict from the RT-qPCR literature. Likely LNA-induced primer-template duplex stabilization and slower dissociation kinetics^63^ hampered the key mechanism of LAMP, namely hairpin-mediated self-foldback and deannealing^11^. This is further supported by our experiments with the E2L set (Fig. 1f) where we varied the *T*_*m*_ of F2/B2 regions and observed improved performance with shorter primers with lower *T*_*m*_ (and presumably affinity). Interestingly, LNA modifications of the B1c/F1c regions of the BIP/FIP primers, either had neutral (F1c) or slightly beneficial (B1c) effects on sensitivity, specificity (i.e., non-specific amplification), and speed that was dependent on the target template. These results extend previous findings^93^ by elucidating the primer selective effects of LNA modifications and help to establish a foundation of principles for further use of LNA in LAMP. They also highlight the potential in revisiting LAMP primer design guidelines as one of the key difficulties with LAMP is the trial-and-error nature of screening in combination with the higher number of priming regions involved. It would also be especially helpful in situations where the target sequence is too short or limited by low complexity base content (high A/T(U) content such as E gene in SARS-CoV-2), making it difficult to design suitable primers. Using LNAs as we demonstrate in our manuscript, may allow for such a set to be designed and used for problematic templates that would otherwise be avoided using classical LAMP primer design principles.

Due to the high amount of amplicon generation, LAMP is well suited towards colorimetric detection. While multiple colorimetric detection systems exist, most LAMP studies use a pH-based system with Phenol Red^45^. It has excellent sensitivity and a perceptible color shift (pink/red to bright yellow), but it does not allow any meaningful reaction buffering in the master mix or sample. This is especially limiting when assaying crude samples, which lack defined composition and contain disparate buffering pH and capacity. Indeed, some groups^22,65,94,95^ have observed samples (and even water) to immediately discolor a Phenol Red-based master mix, whereas others could not utilize buffered sample processing methods as they prevent color change^41,55^. Limiting the input volume of such samples or titrating in an amount of buffer/base to neutralize acidic samples^94^ can be attempted, although this either compromises assay sensitivity or must be optimized on a per-matrix basis. Alternative approaches use pH-independent detectors that permit reaction buffering, including the metallochromic indicators, HNB or Eriochrome Black T (EBT), which exhibit color change in lockstep with the concentration of free Mg^2+^ ^40,96^. However, these dyes suffer from ambiguous color transition and consequently reduced sensitivity.

Our ZBP colorimetric detection system combines the best of both systems: the vivid color transition of Phenol Red with the buffering capacity of HNB (Fig. 4). With its additional advantages (one can optimize Mg^2+^ without affecting colorimetric change and the detection system is unaffected by Mg^2+^/Ca^2+^ in biological matrices) our ZBP colorimetric LAMP test is ideal for difficult samples such as saliva or gargle. A possible disadvantage is that this chemistry might be incompatible with crude sample processing techniques especially those relying on high concentrations of EDTA and/or TCEP (e.g., HUDSON^97^). However, our developed compatible method to inactivate crude gargle specimens while preserving viral RNA (Fig. 5), which requires only basic equipment (block heater, pipettes, mini centrifuge) and takes ~10 min, is comparable with other similar protocols^41,55,65,97^. In clinical validation, this method performed excellent with both gargle specimens and NP swab samples in CDC VTM having concordance between fluorescent- and colorimetric-based results (Supplementary Fig. 6c). Internally, we found it is compatible with saliva too. Another possible caveat is that other ions can form colored complexes with 5-Br-PAPS, which could interfere with the reaction. Cu^2+^ and Fe^2+^ are the most probable interfering ions forming violet complexes with 5-Br-PAPS; however, they are unlikely to be found in high enough concentrations in oral rinse/gargle samples to be an issue. From our early testing, only Fe^2+^ (from blood in input samples) was of potential concern as the Fe^2+^/5-Br-PAPS complex has high stability towards pyrophosphate. This concern was shown to be unfounded because in practice we have not observed any visible interference from blood being present in gargles before processing.

A recent report suggests that Mn^2+^ could be used with 5-Br-PAPS for colorimetric detection of amplification^98^, despite a previous report of this complex not forming under mild conditions^99^ and Mn^2+^ possessing certain theoretical disadvantages such as reduced Bst enzyme fidelity^100^ and inducing mutagenesis during synthesis^101,102^. Intrigued, we conducted a thorough comparison of our ZBP system with the one based on Mn^2+^. In our hands, we observed the Mn^2+^/5-Br-PAPS colorimetric detection system to require an excess of Mn^2+^ and higher pH for the complex to form (Supplementary Fig. 8a), likely explaining why a switch of Zn^2+^ for Mn^2+^ in our ZBP RT-LAMP master mix failed to properly function as a colorimetric detector (Supplementary Fig. 8b). Conducting amplification under the conditions described in the original publication showed the system to function as described; however, we found that using Zn^2+^/5-Br-PAPS instead increased the robustness of colorimetric change with respect to reaction parameters (Supplementary Fig. 8c). Additionally, the Zn^2+^/5-Br-PAPS assay displayed faster amplification (Supplementary Fig. 8d, e) and enhanced sensitivity at low copies/reaction (Supplementary Fig. 8f, g). A more thorough comparison of these two systems is warranted as both might be advantageous under different conditions, like amplification method (e.g., PCR vs LAMP or other isothermal amplification techniques), alternative primer sets (for example, the LAMP primers used by Zhang et al.^98^ are optimized for the Mn^2+^-based system and would likely perform better) or sample input types. With respect to the latter, it will be crucial to identify a direct sample inactivation procedure compatible with the Mn^2+^/5-Br-PAPS system, a task made complicated by the weakly stable Mn^2+^/5-Br-PAPS complex likely being more susceptible to disruption by compounds found in direct samples and inactivation buffers/reagents^99^. Our Zn^2+^-based system and inactivation reagents are uniquely suited towards analytically difficult specimen types, such as gargle samples and nasal swabs in VTM, and provide a solution for POCT sites where a proper diagnostic laboratory is impractical or impossible.

One of the allures of alternative NAAT technologies like RT-LAMP is their potential to combine high sensitivity, specificity, and quick workflows. RT-LAMP sensitivity with direct samples must be high enough to justify its diagnostic use as RAT testing sites are generally easier and cheaper to establish and maintain. Therefore, large-scale use of RT-LAMP during the ongoing COVID-19 pandemic has been relatively limited^103,104^. Our Vivid COVID-19 LAMP showed an analytical LoD of 1 synthetic RNA copy/µl sample input (=8 copies/reaction), placing it among the most sensitive RT-LAMP^105,106^ and even many RT-qPCR tests^107^. With direct gargle samples, the same assay showed 100% PPA for samples with Ct <34 (Supplementary Data 6) and excellent concordance with RT-qPCR. To put this into perspective, virus infectivity experiments from SARS-CoV-2 positive specimens show a large drop-off in infectivity above Ct 30 with almost no specimens being infectious above Ct 35^108–112^, highlighting that our assay should detect the majority of potentially infectious patients. In terms of specificity (NPA), we tested 232 negative samples over three validations and did not observe a single false positive case, a finding that is critical when conducting mass testing in asymptomatic individuals in low prevalence settings. Our Vivid COVID-19 LAMP assay, which accepts non-invasive gargle samples, is ideal for frequent and repeated testing at sites where sensitive and specific SARS-CoV-2 testing is desired for example at borders, airports, companies, or entertainment venues.

Benchmarking our Vivid COVID-19 LAMP assay to three state-of-the-art LAMP tests confirmed our Vivid COVID-19 LAMP assay performed the best on direct gargle samples and was either equivalent or better than the comparator assays on extracted RNA. A comparison of other key features of these assays (Supplementary Data 7) highlights additional advantages of our Vivid COVID-19 LAMP assay, including optional internal and positive controls and a UDG/dUTP master mix to prevent amplicon contamination. Importantly, our assay detects all major VOCs, including the most recent omicron variants.

Since LAMP is rapid, robust, and has minimal equipment requirements, it is well suited for field use and emerging evidence has highlighted its utility in POCT scenarios^9,18,50,66,96,113–117^. Here we report high-throughput POCT testing by leveraging our ultra-sensitive, pH independent colorimetric assay, digitized sample pairing and tracking, semi-automated reaction preparation, and an unbiased, AI-assisted mobile application that interprets and reports colorimetric results into a streamlined mobile laboratory. This flexible workflow enables a small crew (3-4 people) in a single unit to process several hundred samples per day. Indeed, as a testament to the utility of Vivid COVID-19 LAMP for field testing, it has been successfully utilized by conducting 8,733 LAMP tests spanning a variety of real-world testing situations like conferences, concerts, sporting events, private companies and schools, and national public testing (details described in Supplementary Data 8).

In summary, our SARS-CoV-2 assay utilizing multiple LAMP improvements is a simple, rapid (results in 50 min for maximum sensitivity), mobile-ready, and sensitive method that is scalable and complementary to existing testing modalities in use to detect SARS-CoV-2. With a flexible workflow that allows the user to modify reaction speed (and consequently sensitivity), sample format (gargle, saliva, VTM – extracted RNA or direct), and detection endpoint (colorimetric or fluorescence), this test can be fine-tuned depending on the testing requirements (e.g., symptomatic vs asymptomatic, high- vs low-throughput). Future research should address some minor limitations: 1) since our ZBP master mix is based on proprietary commercial enzymes, adapting this assay to use open source enzymes^41,66,93,118–120^ (e.g., Bst large fragment derivatives, MashUp-RT, RTX, etc.) can facilitate scalability by reducing costs, broadening access, and mitigating reagent supply constraints; 2) developing a lyophilized version of our ZBP master mix to eliminate the necessity for cold chain storage and transport^66,119,120^; 3) while we implemented a machine-guided mobile application to help classify color change, it depends on a stable environment provided by a light box. In future work an automated analysis without a stable environment could be implemented that can make system decisions closer to a human operator. We see potential to utilize a neural network not only for landmark (plate corner) localization but also for sample-level localization and classification to automatically extract features required to infer position and class for each sample. Additionally, implementing APIs to flexibly interface with national health agency information systems could further streamline the workflow.

Overall, our Vivid COVID-19 LAMP assay can be a powerful diagnostic tool as we approach the endemic phase of SARS-CoV-2, and the innovations behind this assay can easily be adapted for POCT applications for detecting other pathogens (e.g., malaria, flu, etc.) and preparing for future pandemics.

## Methods

### RT-LAMP

For RT-LAMP, various reaction mixes were used throughout the development and their exact compositions are described in Supplementary Data 1. Individual figure descriptions of experiments explicitly specify the reaction mix used and any changes to reaction mix component concentrations and/or additional additives. Enzymes and specialty proteins were purchased from New England Biolabs (USA), specifically Bst 2.0 WarmStart® DNA polymerase (M0538), WarmStart® RTx Reverse Transcriptase (M0380), Antarctic Thermolabile UDG (M0372), WarmStart® Master Mix (M1800) and Extreme Thermostable Single-Stranded DNA Binding Protein (ET SSB; M2401). In some cases, New England Biolabs pre-formulated reaction-ready master mix was used instead (M1804). All assays utilized either 20 or 50 μl reaction volumes and included a fluorescent intercalating dye (SYTO 9, S34854, Thermo Fisher Scientific, USA; or SYTO 59, S11341, Thermo Fisher Scientific, USA). Unless specified otherwise, final reaction concentrations of LAMP primers were 1600 nM for FIP/BIP, 200 nM for F3/B3, and 600 nM for LF/LB per set and amplification was run at 65 °C on an Agilent AriaMx Real-Time PCR System (G8830A, Agilent, USA).

For SARS-CoV-2 detection in RNA RT-LAMP, positive test reactions utilized template synthetic SARS-CoV-2 RNA mixed with human genomic DNA (COV019, Exact Diagnostics, USA). During ZBP master mix development, 7.5 ng of pure human genomic DNA (G3041, Promega) per reaction was used as the template instead. For direct RT-LAMP reactions, negative gargle samples were spiked with a specified amount of inactivated SARS-CoV-2 virions, NATtrol™ SARS-Related Coronavirus 2 (SARS-CoV-2) Stock (#NATSARS(COV2)-ST, ZeptoMetrix, USA). In direct experiments, inactivated gargle input was 3 µl or 8 µl for 20 µl and 50 µl reaction formats, respectively except for the input volume testing experiment where the inputs spanned 1–5 µl range in a 20 µl reaction format. Real-time amplification was continuously monitored every 30 s by measuring fluorescence in an appropriate optical channel (FAM for SYTO 9, Cy5 for SYTO 59) and the reaction was terminated once the predetermined amount of time elapsed. Reaction duration varied from 40 to 90 min and is either specified in figure legends or is signified in the graphs by the highest time to threshold (TTR) value. Time to threshold was determined by the Agilent Aria 1.7.1 software (Agilent, USA) using custom threshold settings where the threshold was set to 4% of average relative fluorescence intensity of reactions which reached the plateau phase of amplification. LAMP amplification products were considered specific if their melting temperature was within a ± 0.5 °C tolerance of average *T*_*m*_ established for that particular primer set in high template copy number reactions. Images of colorimetric results of LAMP reactions were captured by means of a cell phone camera. Due to high amplification variance at near/sub-limit of detection (LoD) amounts of template, 8 or more replicates per condition were used to better control for this variance and to reduce the impact of artifacts/outliers.

### RT-LAMP primer design and synthesis

The sequences for RT-LAMP primer sets used in this study were either designed from scratch using PrimerExplorer V5 (https://primerexplorer.jp/lampv5/index.html) or adapted from previously published papers^28,55,121^. All primers were designed against the Wuhan reference sequence (NCBI ID: NC_045512.2) and can be found in Supplementary Data 2. Primers were synthesized in-house (MultiplexDX, s.r.o., Slovakia; https://www.multiplexdx.com) and were of HPLC quality or better. All primers had their melting temperatures, GC content, propensity to form homodimers, heterodimers, and secondary structures checked using the IDT OligoAnalyzer™ tool (https://www.idtdna.com/pages/tools/oligoanalyzer), Thermo Fisher Multiple Primer Analyzer tool (https://www.thermofisher.com/sk/en/home/brands/thermo-scientific/molecular-biology/molecular-biology-learning-center/molecular-biology-resource-library/thermo-scientific-web-tools/multiple-primer-analyzer.html), and the mFold server (http://www.bioinfo.rpi.edu/applications/mfold). Both canonical (-TTTT-) and non-canonical linkers were used in FIP/BIP primer design if necessary to disrupt predicted secondary structures and dimers as well as to prevent unintentional extension of F2/F1c and B2/B1c regions at their junctions. In some cases, we also incorporated LNA-modified nucleotides into select primers to test and modify *T*_*m*_ and binding characteristics. Unless specified otherwise, LNAs were incorporated using general guidelines established for PCR primers^62^, e.g., targeting 5’ end of primers and avoiding LNA Gs and especially Cs due to their unnaturally strong base pairing conducive to mishybridization and dimer formation. These general principles have been previously shown by our group to improve binding characteristics and specificity for detecting SARS-CoV-2^82,122,123^.

### Zn^2+^/5-Br-PAPS detection system development

Spectrophotometry of Zn^2+^/5-Br-PAPS solutions was performed as follows. The pyrophosphate concentration-dependent effect on the color of the complex of Zn^2+^ (supplied in the form of ZnSO_4_) with 5-Br-PAPS (93832, Sigma-Aldrich, Germany) was analyzed spectrophotometrically on a Tecan Infinite M1000 Pro Multi-mode Reader (Tecan, Switzerland) by performing a spectral analysis in the 330–600 nm wavelength range while visual color changes were recorded by a cell phone camera. To obtain the spectral analysis curves, 250 µl of mock reaction mix containing base LAMP components (20 mM Tris-HCl pH = 7.9, 20 mM KCl, 60 mM guanidinium chloride, 9 mM MgSO_4_, 0.1% v/v Tween-20, 0.05% v/v Triton X-100, 100 µM 5-Br-PAPS, 50 µM ZnSO_4_) and varying concentrations of sodium pyrophosphate (0–5 mM) were prepared and immediately analyzed.

To study the effects of buffered samples on Zn^2+^/5-Br-PAPS complex color stability, a similar setup as described above was used but with small differences. The same mock reaction mix was prepared (with 0 or 4 mM sodium pyrophosphate) but in such a way that 25% of the final volume was composed of different buffers (pH = 5, 6 – 10 mM sodium citrate; pH = 7, 8, 9–10 mM Tris-HCl; 2.5 mM final concentration) representing common buffering compounds and pH values encountered when analyzing direct samples.

For the set of experiments describing the development of the Zn^2+^/5-Br-PAPS-based colorimetric detection system, only regular DNA LAMP was used. Specifically, LAMP amplification reactions (all 20 µl final volume) were performed in a reaction buffer containing 20 mM Tris-HCl pH = 7.9, 20 mM KCl, 60 mM guanidinium chloride, 1.4 mM dNTPs, 0.1% v/v Tween-20, 0.05% v/v Triton X-100, 500 nM SYTO 59, 100 µM 5-Br-PAPS and 50 µM ZnSO_4_, 320 U/ml Bst 2.0 WarmStart® DNA polymerase and LAMP primer set RNc2 targeting human RPP30 (Supplementary Data 2). Primer concentrations used were identical to those described for RT-LAMP. In an experiment comparing the newly developed colorimetric system to a pH-based colorimetric system, 5-Br-PAPS and ZnSO_4_ were replaced with 100 µM of Phenol Red and different levels of Tris-HCl buffering were used. Each reaction contained 7.5 ng of pure human genomic DNA (G3041, Promega, USA) as the template and the reactions were run at 65 °C on an Agilent AriaMx Real-Time PCR System. Color change was monitored at predetermined intervals during a 30-min reaction duration.

### Gargle specimen collection and inactivation

The final inactivation procedure was as follows: pharynx gargle samples were collected by gargling 5 ml of isotonic sodium chloride solution 3 times for 5 s each. Collected samples were mixed with a 10 × Inactivation buffer containing 100 mM sodium citrate pH = 6, 3 mM TCEP (Tris(2-carboxyethyl)phosphine) hydrochloride, 100 µg/ml carrier RNA (10109223001, Sigma-Aldrich, Germany), and 4.5 mg/ml Pronase (537088, Sigma-Aldrich, Germany) in a 9:1 ratio and incubated at RT for 3 min. Thereafter, the samples were inactivated by heating at 95 °C for 7 min. Inactivated samples were briefly (30–60 s) centrifuged on a benchtop mini-centrifuge (FC5306, OHAUS, Germany) to pellet insoluble material and the supernatant was used as input for direct RT-LAMP. Additionally, during inactivation procedure development, variable times, temperatures, and concentrations of selected reagents (Pronase, TCEP, Triton X-100) were tested as specified in the relevant experimental figures.

### In silico inclusivity and cross-reactivity analyses

We downloaded 500 sequences for SARS-CoV-2 variants (Alpha, Beta, Gamma and Delta) and 4500 for SARS-CoV-2 Omicron variant from the GISAID repository (accessed on March 29^th^ 2022) and aligned them to the Wuhan reference sequence (NCBI ID: NC_045512.2) using the MAFFT alignment tool (with the parameter - auto)^124^. Next, we filtered out sequences containing ambiguous bases in target primer regions (details can be found on Github (https://github.com/MultiplexDX/corona_cheks)^82^) and called the 90% consensus sequences using SeaView^125^. For in silico cross-reactivity testing, we blasted our LAMP primers against NCBI annotated sequences (GenBank®) selected from a list of high priority pathogens from the same genetic family of SARS-CoV-2 and organisms likely to be present in respiratory/saliva samples (Supplementary Data 3). The B1c/F1c and B2/F2 regions of BIP and FIP, respectively, were blasted as separate primers. Primers that contained ≥80% homology with a given microorganism were recorded for further inspection and in some circumstances wet-lab cross-reactivity testing.

### Wet-lab inclusivity testing

SARS-CoV-2 variant reactivity was confirmed on both commercial whole viral genome positive controls as well as on patient samples with confirmed lineage status by sequencing. Commercial controls from Twist Bioscience (USA) containing Wuhan-Hu-1 (102024, Control 2), Alpha (103907, Control 14), Beta (104043, Control 16), Gamma (104044, Control 17), Delta (104538, Control 28), Omicron BA.1 (105204, Control 48) and Omicron BA.2 (105345, Control 50) variants were first compared against positive control from Exact Diagnostics (used to determine LoD of the developed assay) using RT-qPCR (rTEST COVID-19 Allplex qPCR, MultiplexDX s.r.o., Slovakia) and were subsequently diluted to identical Ct values. Viral RNA stocks adjusted in this manner were used to test Vivid COVID-19 LAMP variant reactivity at 10 ×, 5 × and 3 × LoD concentration as determined during assay development (LoD = 1 cp/µl with RNA as input) with 8 replicates per multiple of LoD.

To test reactivity against real patient samples with sequencing-confirmed lineage status, extracted RNA from Alpha (3 samples), Beta (2 samples), Delta (3 samples), Omicron BA.1 (3 samples), Omicron BA.2 (3 samples) Omicron BA.2.12.1 (3 samples), Omicron BA.4 (3 samples) and Omicron BA.5 (3 samples) patient samples (Supplementary Data 5) first had their Ct checked by RT-qPCR (rTEST COVID-19 Allplex qPCR, MultiplexDX s.r.o., Slovakia) and were diluted to a target Ct range of 31–33. After confirming the target range has been reached with RT-qPCR, a single replicate per sample was assayed with Vivid COVID-19 LAMP.

### Wet-lab cross-reactivity testing

To evaluate primer set cross-reactivity to other closely related coronaviruses, we used the „Coronavirus RNA specificity panel“ (011N-03868, European Virus Archive – Global; EVAg), which contains RNA viruses HCoV-229e, HCoV-OC43, HCoV-NL63, MERS-CoV, and SARS-CoV-2, each in a separate tube. To assess cross-reactivity to non-coronavirus respiratory viruses, we tested a set of respiratory viruses (AmpliRun® PCR controls; Vircell Microbiologists, Spain) containing RNA of Influenza A H1N1 (MBC028), Novel Influenza A H1N1 (MBC082), Influenza A H3N2 (MBC029), Influenza A H5N1 (MBC052), Novel Influenza B (MBC030), Human parainfluenza (MBC105), Respiratory syncytial virus (MBC041) and Human rhinovirus (MBC091), each provided in a separate tube. LAMP assays were performed in 3 replicates for each of the indicated viruses in an amount of 3/6 µl for EVAg standards (unknown concentration) and 1000/2000 copies per reaction for Vircell standards for ZBP RNA RT-LAMP and Vivid COVID-19 LAMP, respectively.

### Clinical sample processing and validation

All clinical validations were performed by a blinded experimenter at Biomedical Research Center, Institute of Virology, Slovak Academy of Sciences (BMC-SAS) and both the index and evaluated tests were performed at the same time minimizing any possible evaluation bias. The specimens obtained from participants were part of routine SARS-CoV-2 testing by the Biomedical research Center of the Slovak Academy of Sciences and therefore were not subject to any recruiting inclusion or exclusion criteria. Thus, enrollment of participants and selection of specimens was random and not subject to biases that could impact the results. All identifiable information about participants was anonymized. Validations were conducted on distinct samples that were freshly collected and no samples were measured repeatedly. All experiments and clinical validations involving collection and use of human specimens were reviewed and approved by the Ethics committee of the Biomedical research Center of the Slovak Academy of Sciences, Bratislava, Slovakia (Ethics committee statement No. EK/BmV-02/2020) and were performed according to their regulations and guidelines. Informed consent was obtained from all subjects.

Clinical performance of RNA SARS-CoV-2 ZBP RT-LAMP was conducted using a selected set of 139 SARS-CoV-2 positive samples (81 NP swabs eluted in CDC viral transport medium (VTM) and 58 isotonic saline gargle samples) and 111 negative samples (48 NP swabs eluted in CDC VTM and 63 isotonic saline gargle samples). CDC VTM was prepared according to the official CDC instructions (https://www.cdc.gov/coronavirus/2019-ncov/downloads/Viral-Transport-Medium.pdf). Viral RNA was extracted using the RNAdvance Viral Kit (Cat. No. C63510; Beckman Coulter, USA) and the Biomek i5 Automated Workstation (Beckman Coulter, USA) and then both RNA SARS-CoV-2 ZBP RT-LAMP and the index test (rTEST COVID-19 Multiplex qPCR, MultiplexDX s.r.o., Slovakia) were performed and compared; extracted RNA input was 5 µl for both tests. RNA SARS-CoV-2 ZBP RT-LAMP utilized a triplexed assay in a 20 µl format with 45 min of runtime.

Direct SARS-CoV-2 ZBP RT-LAMP validation was performed with 75 SARS-CoV-2 positive samples (43 NP swabs eluted in CDC VTM and 32 isotonic saline gargle samples) and 56 negative samples (24 NP swabs eluted in CDC VTM and 32 isotonic saline gargle samples). All samples were analyzed either fresh or within 5 days of collection while being stored in a refrigerator. Samples for Direct SARS-CoV-2 ZBP RT-LAMP were processed and inactivated as per the developed procedure for gargle inactivation; NP swabs were processed in the same manner where gargle was replaced by VTM. For the index test (rTEST COVID-19 Multiplex qPCR, MultiplexDX s.r.o., Slovakia), RNA was extracted as described above. Direct SARS-CoV-2 ZBP RT-LAMP inactivated input was 3 µl while extracted RNA input for the index test was 5 µl. Direct SARS-CoV-2 ZBP RT-LAMP utilized a triplexed assay in a 20 µl format with 45 min of runtime.

Vivid COVID-19 LAMP validation was almost identical to Direct SARS-CoV-2 ZBP RT-LAMP with a few differences. The validation included 108 SARS-CoV-2 positive and 65 negative samples all of which were isotonic saline gargle samples. Vivid COVID-19 LAMP used 8 µl of inactivated gargle as input, while the index test (rTEST COVID-19 Allplex qPCR, MultiplexDX s.r.o., Slovakia) used 5 µl of extracted RNA as input. Vivid COVID-19 LAMP utilized a quadruplexed assay in a 50 µl format with 40 min of runtime.

### SARS-CoV-2 virus cultivation

For the series of virus spike-in experiments (see below), we used SARS-CoV-2 cell culture isolate Slovakia/SK-BMC-BA43/2022 which was isolated from a COVID-19 patient from Slovakia in July 2022. The virus belongs to the BE.1.1 lineage (alias of BA.5.3.1.1.1; Omicron VOC) and is deposited in the European virus archive GLOBAL (available at https://www.european-virus-archive.com/virus/sars-cov-2-strain-slovakiask-bmc-ba432022-omicron-voc-be11-alias-ba53111). The virus stock was prepared in a Biosafety level 3 containment laboratory (Biomedical Research Center of the Slovak Academy of Sciences, Slovakia) by infecting Vero E6 cells (Vero C1008, ATCC CRL 1586) cultured in Eagle’s minimal essential medium (EMEM, Lonza, Switzerland) supplemented with 5% FBS (GIBCO, USA), Penicillin-Streptomycin-Amphotericin B Solution (10 ml/l, Lonza, Switzerland).

### Characterization of viral load, reaction runtime, and sensitivity

To establish required runtimes of Vivid COVID-19 LAMP for different levels of input viral load, 96 positive and 24 negative gargle samples were tested. Specifically, 6 different gargle backgrounds were first screened for negativity by the means of a PCR test (rTEST COVID-19 SuperRapid qPCR, MultiplexDX s.r.o., Slovakia). Subsequently, 16 serial 2-fold dilutions of live BA.5 SARS-CoV-2 virions (expected starting Ct 18.68, serially diluted with isotonic saline) were spiked into all 6 gargle backgrounds (10 µl into 200 µl gargle) generating 96 unique positive gargle samples. For negative samples, 4 replicates each of the 6 gargle backgrounds were used. Positive gargle specimens prepared this way had their PCR Ct values experimentally determined (rTEST COVID-19 SuperRapid qPCR, MultiplexDX s.r.o., Slovakia). Finally, gargle samples were processed as per the procedure established for Vivid COVID-19 LAMP and all inactivated sample supernatants were assayed 6 times independently with varying reaction runtimes (17.5/20/25/30/35/40 min). Reaction plates were then cooled to room temperature and imaged in quick succession with a cell phone camera for analysis.

### Performance comparison of multiple SARS-CoV-2 LAMP assays

Sensitivity and specificity of Vivid COVID-19 LAMP, SARS-CoV-2 Colorimetric ReadiLAMP™ Kit (A52539, Thermo Fisher Scientific, USA), SARS-CoV-2 Rapid Colorimetric LAMP (E2019S, New England Biolabs, USA) and SARS-CoV-2 LAMP assay as described by Rabe & Cepko^55^, were compared on ﻿a﻿ ﻿panel of archived RNA samples and direct gargle specimens (non-overlapping 48 positives and 32 negatives per input type) with medium to very low viral loads (Ct range of 29.0–37.6 for RNA and 25.9–35.9 for gargles). RNA was extracted from gargle samples as described in the “clinical sample processing and validation” above and then both archival RNA samples and extracted RNA from gargle specimens was assayed with rTEST COVID-19 Allplex qPCR to obtain reference Ct values. For all assays care was taken to ensure that samples underwent the same number of freeze-thaw cycles and samples were processed as quickly as possible to minimize RNA degradation. All LAMP assays were performed at 65 °C on an Agilent AriaMx Real-Time PCR System (G8830A, Agilent, USA) either in a 25 µl reaction format for 30 min (Colorimetric ReadiLAMP™ Kit, SARS-CoV-2 Rapid Colorimetric LAMP, Rabe & Cepko LAMP assay) or 50 µl reaction format for 40 min (Vivid COVID-19 LAMP). Results were imaged with a cell phone camera using the same background and lighting conditions. Sample inputs were fixed to 5 µl per reaction in the case of extracted RNA experiments while with direct gargle specimens sample input was adjusted based on assay-specific recommendations, specifically 8 µl with Vivid COVID-19, 2 µl with Colorimetric ReadiLAMP™ Kit, 1 µl with SARS-CoV-2 Rapid Colorimetric LAMP and 5 µl with Rabe & Cepko LAMP assay. Additionally, different direct sample inactivation procedures were used to process gargle specimens as suggested/required by the various assays (see below).

For the Rabe & Cepko LAMP assay, custom reagents were prepared in-house (primers, 100 × Lysis buffer) while the commercially available WarmStart® Colorimetric LAMP 2 × Master Mix (DNA & RNA) (M1800S, New England Biolabs, USA) was used as the master mix. Based on preliminary experiments on a group of gargle specimens, the concentration of NaOH in Rabe & Cepko 100 × Lysis buffer was adjusted to 1 N NaOH as this concentration showed good pH neutralizing ability with gargle specimens while still allowing the color change to proceed in positive reactions (final composition of 100 × Lysis buffer was 250 mM TCEP hydrochloride, 100 mM Na_2_EDTA and 1 N NaOH). Gargles were inactivated by mixing 99 µl of gargle with 1 µl of the 100 × Lysis buffer, heated at 95 °C for 5 min and briefly centrifuged (2000 RCF, 30–60 s). Supernatant was subsequently used as input.

For Thermo Fisher Scientific ReadiLAMP™ Kit, direct gargle samples were processed with the most sensitive method as recommended by the manufacturer by mixing 50 µl direct sample and 50 µl of a solution composed of 500 mM TCEP-NaOH pH=8.0 and then heated at 95 °C for 5 min. Supernatant obtained after a brief centrifugation step (2000 RCF, 30–60 s) was used as input.

For NEB Rapid Colorimetric LAMP, untreated gargle samples were used directly as input as no specific inactivation procedure was suggested by the kit manufacturer.

For Vivid COVID-19 LAMP, 90 µl of every gargle was treated as described in this manuscript in the methods subsection “Gargle specimen collection and inactivation”.

### Machine-guided software algorithms, design & evaluation

We have used traditional computer vision techniques^126^ to extract plate positioning and colorimetric results from the photo based on manual feature engineering, which allowed us to circumvent the paucity of training data. To ensure consistent lighting conditions and relative position necessary to image consistent reaction end colors, a 3D printed light box with a fixed drawer and LED lights were constructed. We expected discrepancies in plate positioning caused by human variation, so we utilized the KeypointsNetwork (KpsNet) neural network to look for the four corners of the plate in a photo. This approach, inspired by facial landmark detection^127–129^, efficiently localizes all corners of a plate both in and outside of a light box. Subsequently, the plate is cropped and aligned (perspective transform) from the photo with results overlayed on top for manual validation by a human operator.

To achieve sample-level classification post-transform, the system segments and classifies each sample based on color saturation to eliminate uninteresting pixels, which could have a deleterious impact on further classification process. The classification is based on hue value frequency for each pixel in the sample. To be able to assign hue pixel value to a specific class, we defined hue color value ranges from the HSV color space corresponding to all possible classes permitted by the stable environment of the light box.

To train the neural network, we prepared a custom dataset consisting of video frames in which the plate is captured from various viewpoints and in various positions, and then manually annotated the four corners of a plate in each frame (approx. 1800 frames). For annotations, we used open-source annotation tools (CVAT). To ensure real time experience the pretrained MobileNet V2^130^ was used as the backbone, which has considerably fewer parameters and smaller computational complexity than other popular backbones such as AlexNet, VGG, ResNet, Inception.

Performance of the final algorithm was evaluated on a test dataset derived from National Public Slovak COVID-19 testing consisting of 4398 50 µl SARS-CoV-2 reactions (2449 negative, 141 inconclusive, and 1808 positive results) and 4400 25 µl RNase P reactions (221 negative, 97 inconclusive, and 4082 positive results). These numbers are slightly different from those reported in Supplementary Data 8 due to the inclusion of control reactions, some reaction duplicates, reactions of non-registered tests (i.e., tests of the testing personnel), reactions with no input but suitable for color discrimination, tests classified as invalid for reporting (double negatives), and tests which could not be linked with official governmental patient data due to technical errors at the time of testing. Performance was analyzed separately for the two different reaction types as they have different physical characteristics (differences in volume and by extension HSV color parameters). Final data are presented as 3×3 confusion matrices comparing expected class results (as judged by a human operator) to those determined by the algorithm on a frequency distribution basis. Raw numerical data used to construct the final confusion matrices can be found in Supplementary Data 9.

### Materials & reagents

Source of basic materials and reagents not specified in relevant method sections were sourced from either Merck (Germany), Sigma-Aldrich (Germany), Thermo Fisher Scientific (USA), Lonza (Switzerland), or CentralChem (Slovakia). All reagents were of at least *p.a*. quality if such was commercially available, otherwise reagents of molecular biology / cell culture grade were used instead.

### Statistics and reproducibility

All clinical data were analyzed in GraphPad Prism 9.0. Icons used in Fig. 6d are public domain icons (CC0 1.0 Universal (CC0 1.0) Public Domain Dedication) obtained from https://www.labicons.net/ and http://www.clker.com/. Percent positive agreement (PPA) and negative percent agreement (NPA) values were obtained using contingency table analysis from RT-qPCR and RT-LAMP results. For specific Ct ranges and/or sample types, the same analysis was performed but only with samples matching the description of the subgroup. In all cases 95% confidence intervals were calculated using the Wilson/Brown method. For figures with regular bar charts and scatter plots, averages represent arithmetic means and error bars standard errors of the mean. In general, 8 to 24 technical replicates were used per group during test development. For clinical validation and VOC testing from patient samples, 1 technical replicate per 1 biological replicate was tested. Replicate information is further specified in individual figure legends. For some camera plate images that had variable lighting we made minor whole-image adjustments to brightness and/or contrast to improve clarity of reaction colors within the same experiment.

### Reporting summary

Further information on research design is available in the Nature Portfolio Reporting Summary linked to this article.

## Supplementary information

Peer Review File

Supplementary info

Description of Additional Supplementary Files

Supplementary Data 1-9

Supplementary Data 10

Supplementary Video 1. Software and mobile lab showcase

Reporting summary

## Data Availability

The custom code used for the machine-guided classification of colorimetric results is provided as a supplement and can also be downloaded freely at https://github.com/MultiplexDX/LAMP-extractor. At the same Github page, there is also an executable script for Windows PowerShell, input plate images and corresponding human operator classifications, and a README.txt file containing user instructions and other details, which allows the user to verify the underlying results of the machine-guided color classification (Fig. 10 and Supplementary Data 9).

## Data availability

The data that support the findings of this study are available within the paper and its supplementary information files. The source data used to construct the tables and figures is provided in Supplementary Data 10. The underlying data may also be obtained from the corresponding authors upon reasonable request.
