# Supplementary material for: Vivid COVID-19 LAMP is an ultrasensitive, quadruplexed test using LNA-modified primers and a zinc ion and 5-Br-PAPS colorimetric detection system
Source: Commun Biol. 2023 Mar 2;6:233. doi: 10.1038/s42003-023-04612-9 (PMC9979146; doi:10.1038/s42003-023-04612-9)
Supplement: Supplementary file 2 — Supplementary info [file 42003_2023_4612_MOESM2_ESM.pdf]

Supplementary Information

Vivid COVID-19 LAMP is an ultrasensitive, quadruplexed test using LNA-modified primers and a zinc ion and 5-Br-PAPS colorimetric detection system

Adrián Szobi, Katarína Buranovská, Nina Vojtaššáková, Daniel Lovíšek, Halil Önder Özbasak, Sandra Szeibeczederová, Zuzana Hudáčová, Viera Kováčová, Diana Drobná, Piotr Putaj, Stanislava Bírová, Ivana Čirková, Martin Čarnecký, Peter Kilián, Peter Jurkáček, Viktória Čabanová, Kristína Boršová, Monika Sláviková, Veronika Vaňová, Boris Klempa, Pavol Čekan, Evan D. Paul

Supplementary Fig. 1.

a

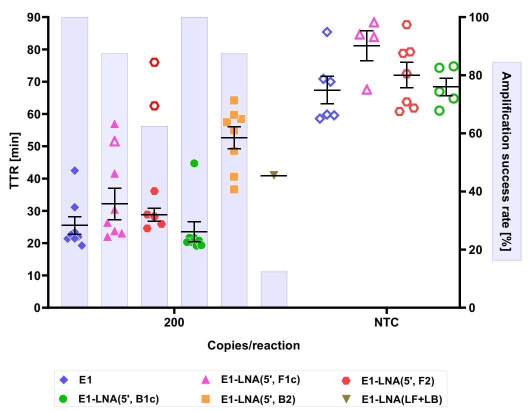

FIP
F1c: 5'-CGC+AG+TAAGGATGGCTAGTGT-3'
F2: 5'-+A+TAGCG+TACTTCTTTTTCTTGC-3'

BIP
B1c: 5'-CT+TCGATTGTGTGCGTACTGCTG-3'
B2: 5'-T+T+TCAG+A+TTTTTAACACGAGAG-3'

LF: 5'-AC+T+AGC+AAGATAACCACGAA-3'
LB: 5'-CG+TG+AGTCTTGTAAAACCTTC-3'

b

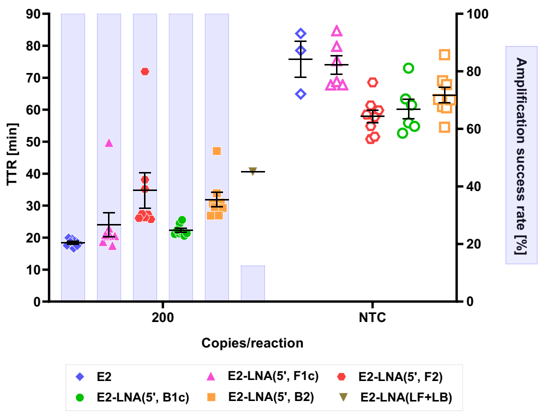

FIP
F1c: 5'-CGC+AG+TAAGGATGGCTAGTGTA-3'
F2: 5'-AC+AGGT+ACGTTAATAGTTAATAGCG-3'

BIP
B1c: 5'-G+TG+TGCGTACTGCTGCAAT-3'
B2: 5'-+T+TCAG+A+TTTTTAACACGAGAG-3'

LF: 5'-CC+ACG+A+AAGCAAGAAAAAGAAGT-3'
LB: 5'-CG+TG+AG+TCTTGTAAAACCTTCT-3'

c

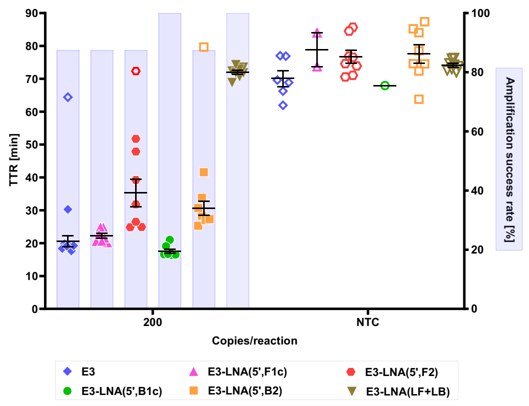

FIP
F1c: 5'-CGC+AG+TAAGGATGGCTAGTGTA-3'
F2: 5'-GCG+T+ACTTCTTTTTCTTGCTT-3'

BIP
B1c: 5'-C+T+TCGATTGTGTGCGTACTGC-3'
B2: 5'-CG+AGAGTAAACGTAAAAAGAAGGT-3'

LF: 5'-+AC+T+AGCAAGAATACCACGA-3'
LB: 5'-+AC+T+AGCAAGAATACCACGA-3'

Supplementary Fig. 1. Locked nucleic acids in inner primers and loop primers.

a-c Effects of LNA-modifications in individual priming regions of inner primers (F1c, F2 in FIP and B1c, B2 in BIP) and loop primers (LF/LB). Modifications showed detrimental effects on the performance of E1 **a**, E2 **b**, E3 **c**, such as lower number of amplified replicates, increased amplification variance, higher TTRs and frequency of non-specific amplification.

Open symbols represent non-specific products, whereas closed symbols are specific as determined by melt curve analysis of amplification products. Highest time to reaction value on y-axis is equal to the total duration of the reaction. All reactions were performed using "NEB WarmStart" reaction mix. "+N" represents a locked nucleic acid base. Error bars represent standard error of the mean. NTC – no-template control; TTR – time to reaction.

Supplementary Fig. 2.

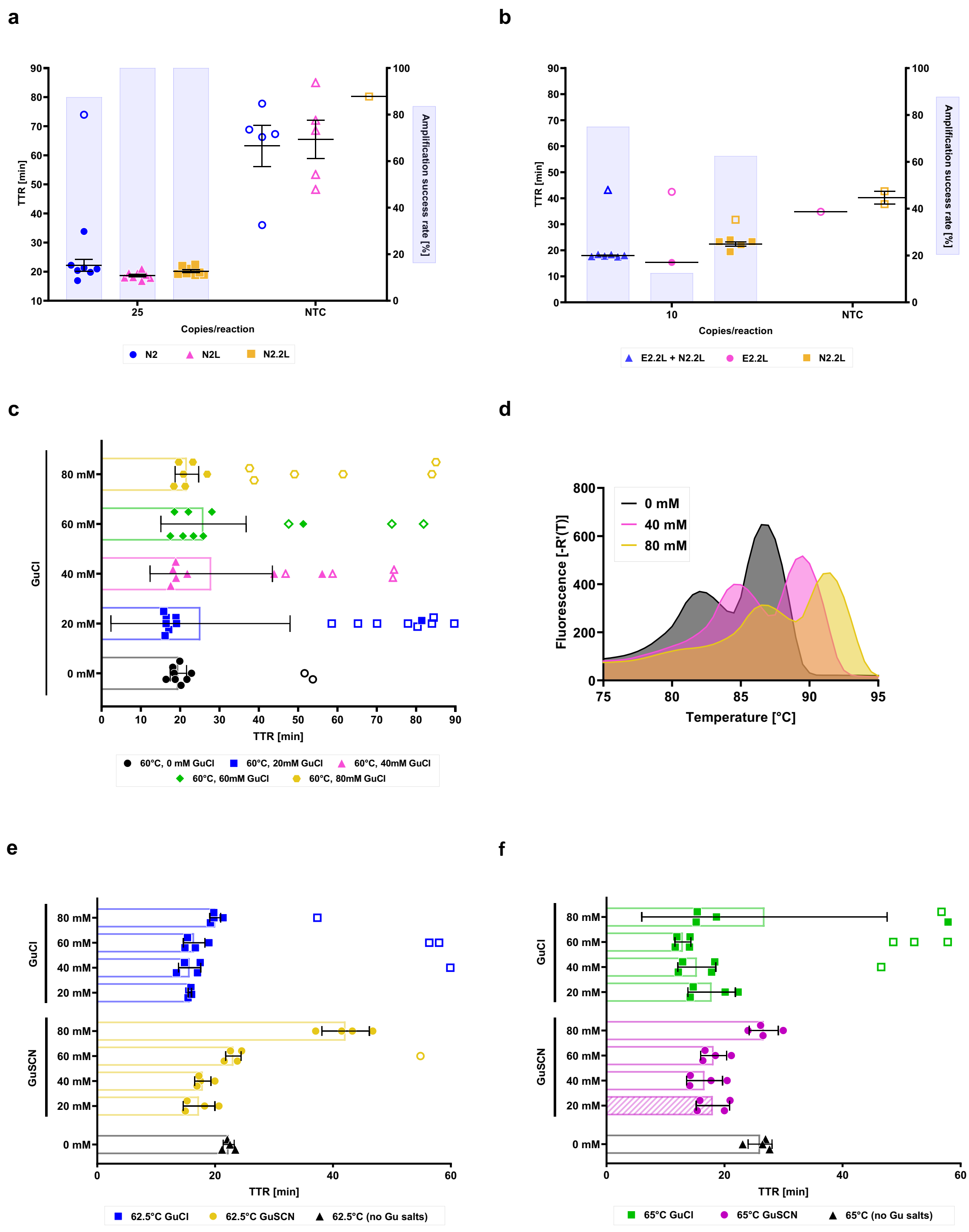

Supplementary Figure 2. Primer set screening for the detection of SARS-CoV-2 N gene, duplexed detection of N and E genes, and further reaction optimizations of guanidinium salts.

a Performance of the N2 primer set and its modified variants for the detection of SARS-CoV-2 N gene. The figure shows the N2 primer set, whose sequence was further adjusted in N2L (LNAs in outer primers), and N2.2L (extra FIP linker) primer sets.

b Effects of duplexing of finalized primer sets for E gene (E2.2L) and N gene (N2.2L).

c The figure shows the results of increasing GuCl concentrations on amplification characteristics of a duplexed E2.2L + N2.2L reaction at 60°C using 10 copies of RNA/reaction.

d The peaks demonstrate the effects of increasing GuCl concentrations on melt curves of reaction products of a duplexed E2.2L + N2.2L reaction using 10 copies of RNA/reaction.

e-f Comparison of GuCl and GuSCN salts at reaction temperatures 62.5°C **e** and 65°C **f** using 10 copies of RNA/reaction. The patterned bar highlights the condition chosen for further experiments; corresponds to “NEB WarmStart 1.2” reaction mix.

Open symbols represent non-specific products, whereas closed symbols are specific as determined by melt curve analysis of amplification products. The highest time to reaction value on y-axis **a-b** or x-axis **c, e-f** signifies the total duration of the reaction. Amplification success rate shows the percentage of samples that amplified over the course of the reaction. For NTC reactions PCR-grade water was used as the input. All experiments were performed using the “NEB WarmStart 1.1” reaction mix with additional specified additives, except for **e-f**, which used the “NEB WarmStart 1.2” reaction mix. Error bars represent standard error of the mean. TTR – time to reaction; NTC – no-template control; GuCl – guanidininum chloride; GuSCN – guanidinium isothiocyanate.

Supplementary Fig. 3.

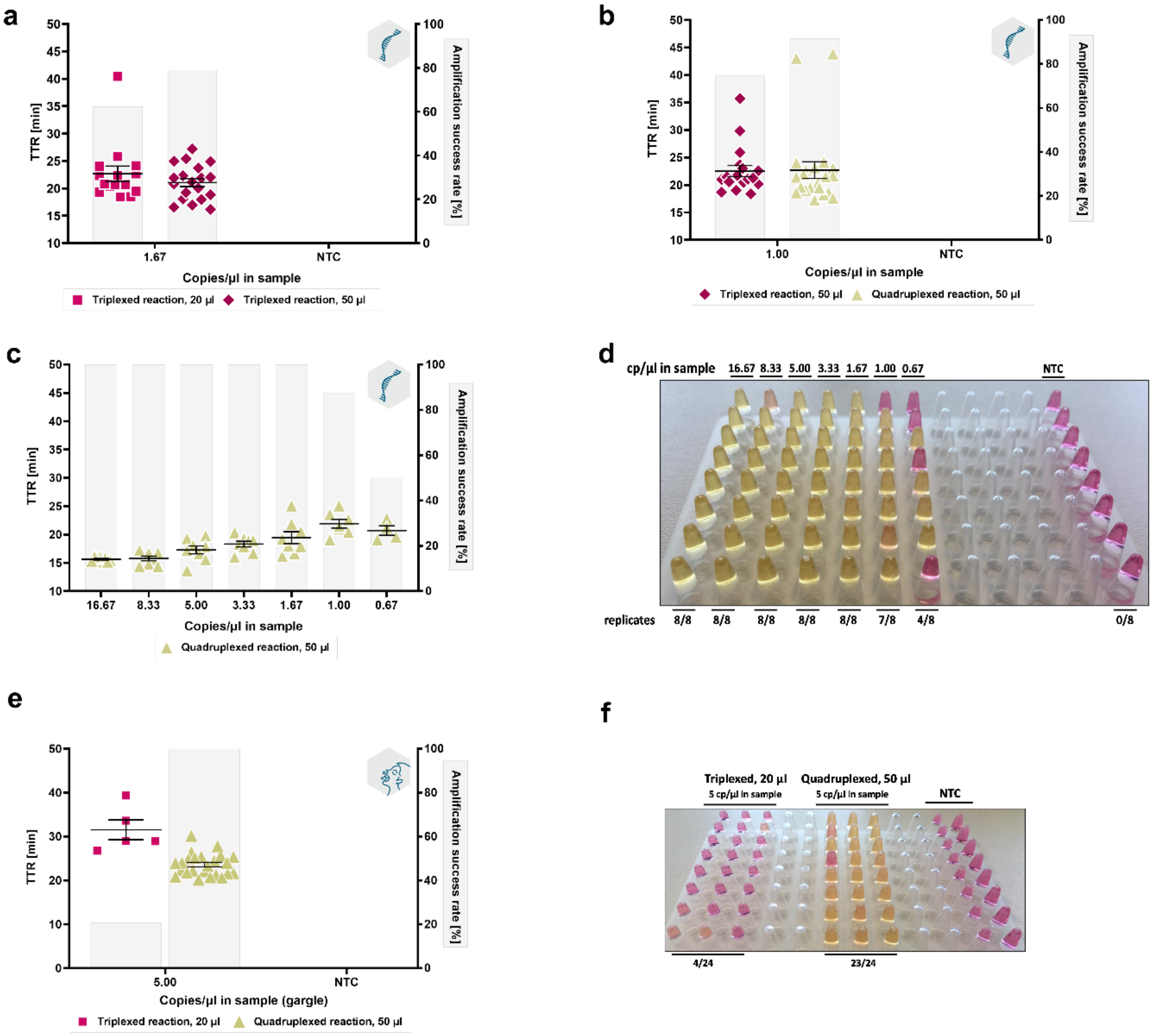

Supplementary Fig. 3. Additional performance comparisons between triplexed and quadruplexed versions of the assay.

a Fluorescence amplification data comparing the performance of 20 and 50 µl reaction volumes utilizing the same triplexed primer mixes. n=24 technical replicates were used for groups with SARS-CoV-2 RNA template while n=8 technical replicates were used for NTC reactions instead.

b Effects of adding another LAMP primer set, As1.2L, into the triplexed LAMP primer set mix in a 50 µl reaction format. n=24 technical replicates were used for groups with SARS-CoV-2 RNA template while n=8 technical replicates were used for NTC reactions instead.

c-d Limit of detection of the quadruplexed 50 µl SARS-CoV-2 assay in the RNA RT-LAMP format. Input sample template concentrations correspond to 50/25/15/10/3/2 copies/reaction in the original triplexed 20 µl reaction format. n=8 technical replicates per group were used.

e-f Performance comparison of the original 20 µl triplexed reaction and 50 µl quadruplexed reaction with gargle specimens spiked with a given concentration of inactivated SARS-CoV-2 virions.

ZBP 1.1G and ZBP 2.0 reaction mixes were used for 20 and 50 µl reaction formats, respectively. Sample input volume was kept roughly proportional to the final reaction volume (3 µl in 20 µl and 8 µl in 50 µl). The highest time to reaction value on y-axis equals the total duration of the reaction. The experiments where we used synthetic RNA as a positive control are marked with a single stranded RNA symbol in the upper right corner; whereas experiments where we used gargle with spiked SARS-CoV-2 virions are marked with a gargling person symbol in the upper right corner. Amplification success rate represents the percentage of samples that amplified over the course of the reaction. n=24 technical replicates were used for groups with SARS-CoV-2 RNA template while n=8 technical replicates were used for NTC reactions instead.

Error bars represent standard error of the mean. NTC – no template reaction; TTR – time to reaction; cp/µl – copies per microliter.

Supplementary Fig. 4.

Positive samples

25.68	25.16	25.83	25.64	26.14	25.09	36.92	35.68	37.39	34.60	36.22	34.12
23.47	23.42	25.40	24.27	24.80	25.79	34.08	34.77	35.84	34.40	34.54	35.14
22.20	23.52	24.03	23.27	24.88	24.10	33.03	32.87	33.32	33.25	32.57	33.38
22.60	21.30	23.04	24.39	22.42	23.71	32.33	31.09	32.04	31.27	31.34	33.04
20.29	20.71	21.54	22.38	21.33	21.98	29.70	28.71	32.01	29.96	32.67	30.92
21.58	20.52	21.95	20.50	21.00	20.44	28.61	27.62	30.32	29.06	29.78	28.89
20.07	18.90	20.05	20.49	19.41	20.49	28.24	28.17	27.00	29.23	27.24	26.81
18.75	17.83	17.97	19.47	20.86	19.48	27.15	26.68	26.24	27.71	27.62	26.66

SARS-CoV-2 PCR Ct results

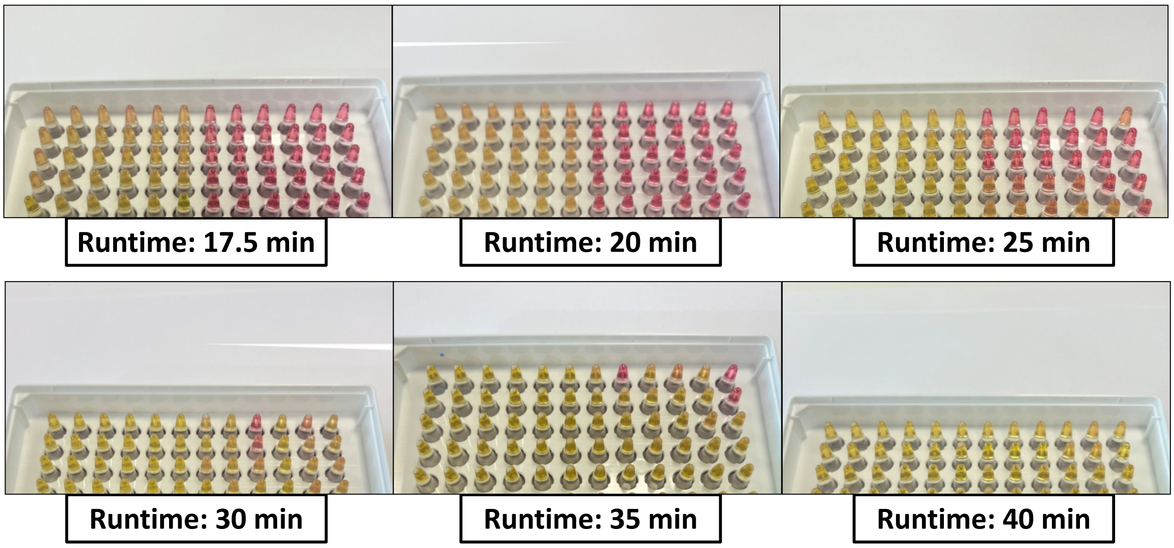

Negative samples

No Ct	No Ct	No Ct	No Ct	No Ct	No Ct
No Ct	No Ct	No Ct	No Ct	No Ct	No Ct
No Ct	No Ct	No Ct	No Ct	No Ct	No Ct
No Ct	No Ct	No Ct	No Ct	No Ct	No Ct

SARS-CoV-2 PCR Ct results

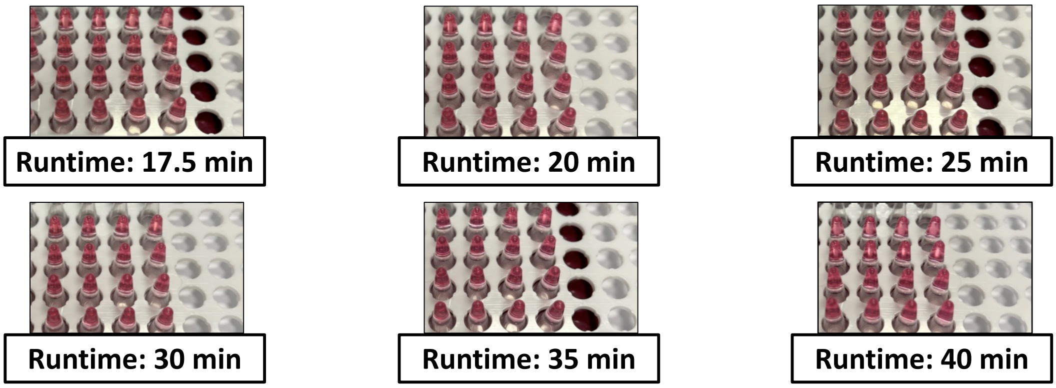

Supplementary Fig. 4. Colorimetric results of time-course/viral load experiment.

Gradient color-coded plate maps (white – lowest Ct; red – highest/no Ct) show reference SARS-CoV-2 Ct values for individual wells as obtained by RT-qPCR. Photos demonstrate progressively higher proportion of positive replicates among higher Ct values with longer amplification times while for negative samples no effect is seen. BA.5 Omicron-spiked positive gargle samples were loaded in such a way that samples have increasing Cts from bottom-to-top in two half-row groups. Every half-row (6 samples) represents 6 different gargle backgrounds spiked with the same theoretical load of SARS-CoV-2 virions. Negative samples of the 6 gargle backgrounds were loaded in quadruplicate within a column instead.

Supplementary Fig. 5.

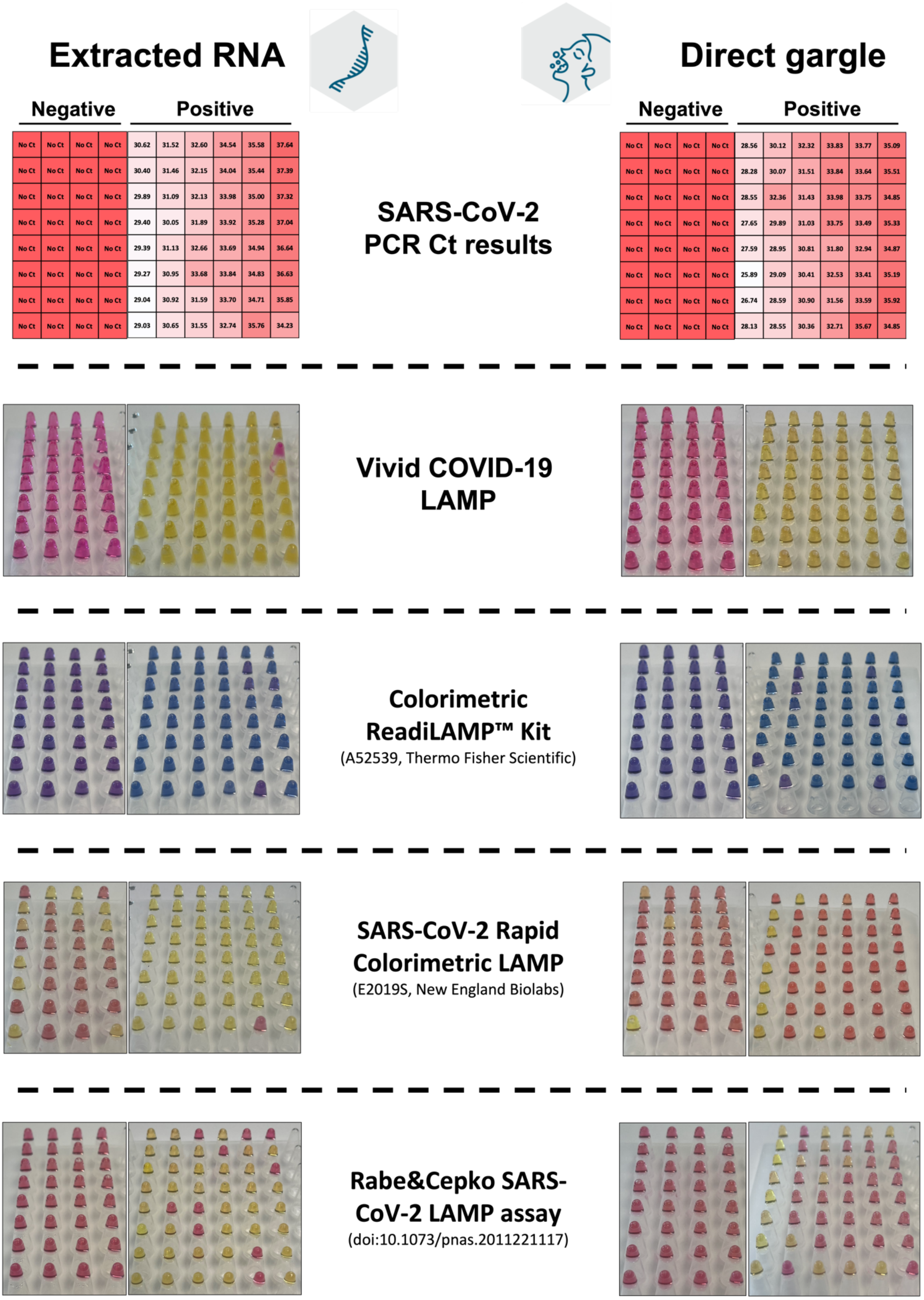

Supplementary Fig. 5. Colorimetric results of compared LAMP SARS-CoV-2 assays.

Gradient color-coded plate maps (white – lowest Ct; red – highest/no Ct) at the top show reference SARS-CoV-2 Ct values for individual wells as obtained by RT-qPCR. Photos show the resulting reaction mix colors after the amplification phase has ended. Left set of photos/plate map correspond to samples with extracted RNA as input while the right side is from positive patient gargle samples. Positive samples were ordered by viral load as measured by RT-qPCR, roughly from top-to-bottom within columns wrapping around to the next column resulting in an average Ct gradient in a left-to-right direction.

Supplementary Fig. 6.

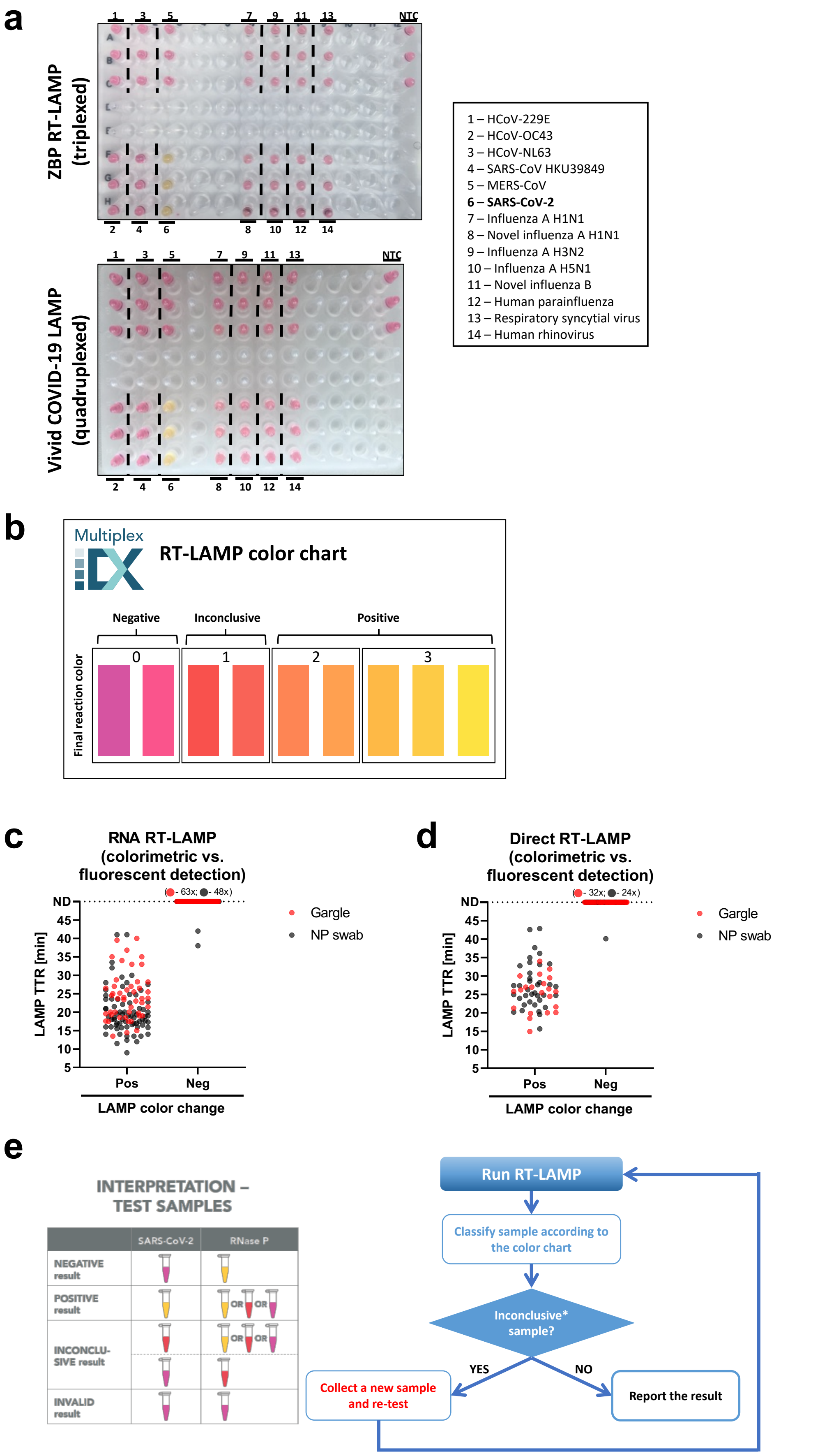

Supplementary Fig. 6. Cross-reactivity wet-lab testing, supplementary clinical validation data.

a Visual results of wet-lab cross-reactivity testing of selected viral pathogens.

b Reference color chart used during clinical validation to classify colorimetric results. Corresponds to the final color chart for reaction color classification of Vivid COVID-19-LAMP.

c-d Concordance between colorimetric and fluorescent detection of LAMP amplification for RNA SARS-CoV-2 ZBP RT-LAMP **c** and Direct SARS-CoV-2 ZBP RT-LAMP **d**.

e Decision tree and combined reaction interpretation for Vivid COVID-19 LAMP test interpretation with an internal control reaction (RNase P) included.

For all patient samples 1 technical replicate per 1 biological replicate was tested.

Supplementary Fig. 7.

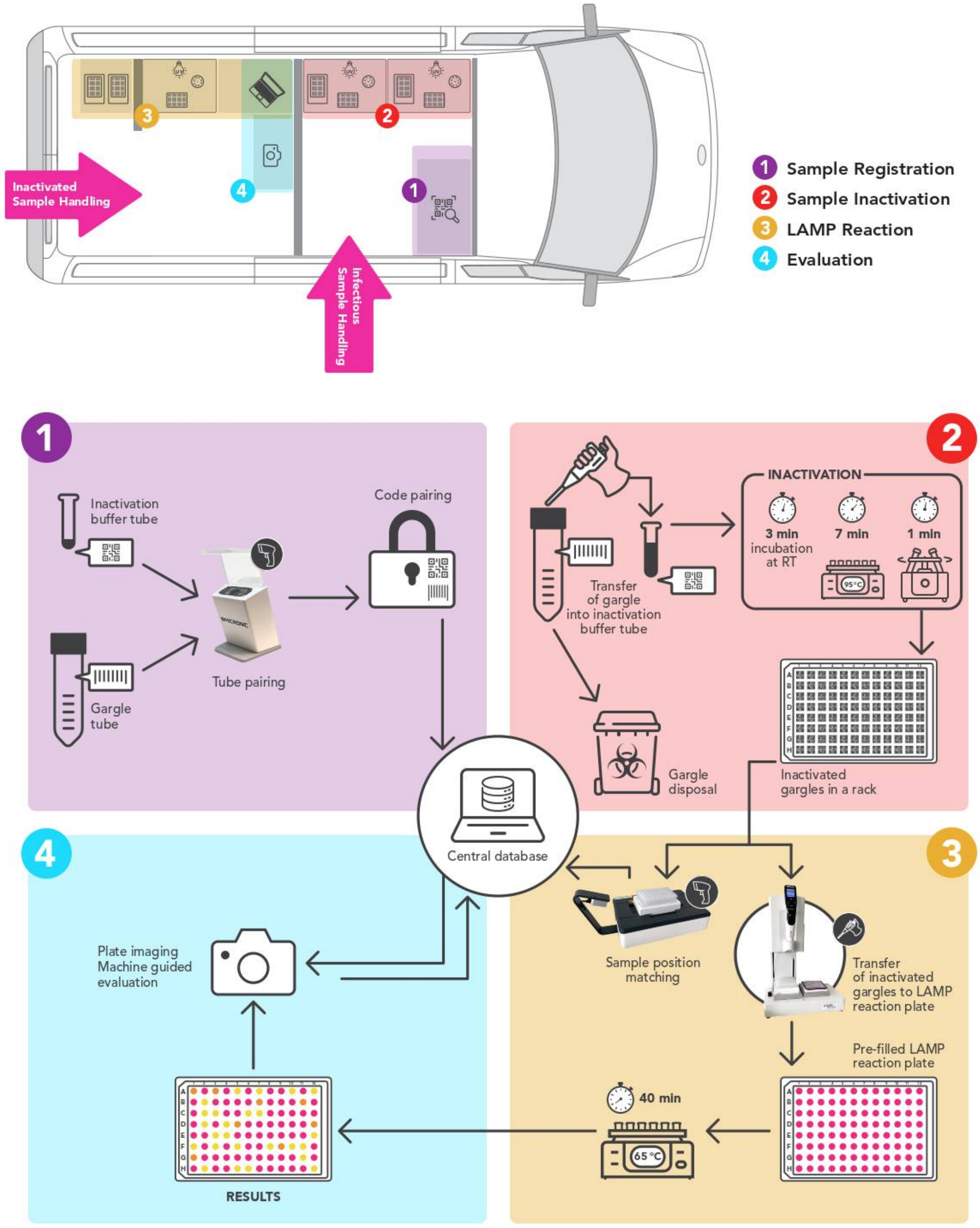

Supplementary Fig. 7. Schematic depiction of mobile LAMP-testing solution.

The mobile laboratory floorplan presented is configured for handling infectious samples. The vehicle (mobile laboratory) is physically divided into two parts based on sample processing status and its infectious potential. Color coding matches workflow processes (sample registration, sample inactivation, LAMP reaction, evaluation) to their spatial arrangement in the vehicle. Both sections of the vehicle have their dedicated air recirculation and filtration systems suitable to maintain Biosafety level 3 environment.

Sample is registered by scanning the barcode on gargle sample tube and the 2D data matrix code on the tube containing inactivation buffer with a code reader. These two codes are saved as a pair in the central database. Samples are then inactivated as per the procedure developed for Vivid COVID-19 LAMP test. Tubes containing inactivated gargles are then put in 96-well racks which are scanned by a specialized rack reader, allowing the database to match sample code to the position in plate. The inactivated gargles are pipetted into PCR plates pre-filled with LAMP reaction mix with a possibility of using high-throughput pipetting tools. After allowing the plate to amplify at 65°C for 40 minutes, the finished plate with color changes is imaged and evaluated with the help of a machine-guided approach. Final results are stored in the central database for further use. Software usage and demonstration of key steps can be viewed in Supplementary Video 1.

Symbol legend: UV bulb – PCR box, code with magnifying glass – tube pairing station, circular object with holes – benchtop mini-centrifuge, rectangle with codes – tube racks, double rectangle with holes – dry block heater or end-point PCR machine, camera – imaging station.

Supplementary Fig. 8.

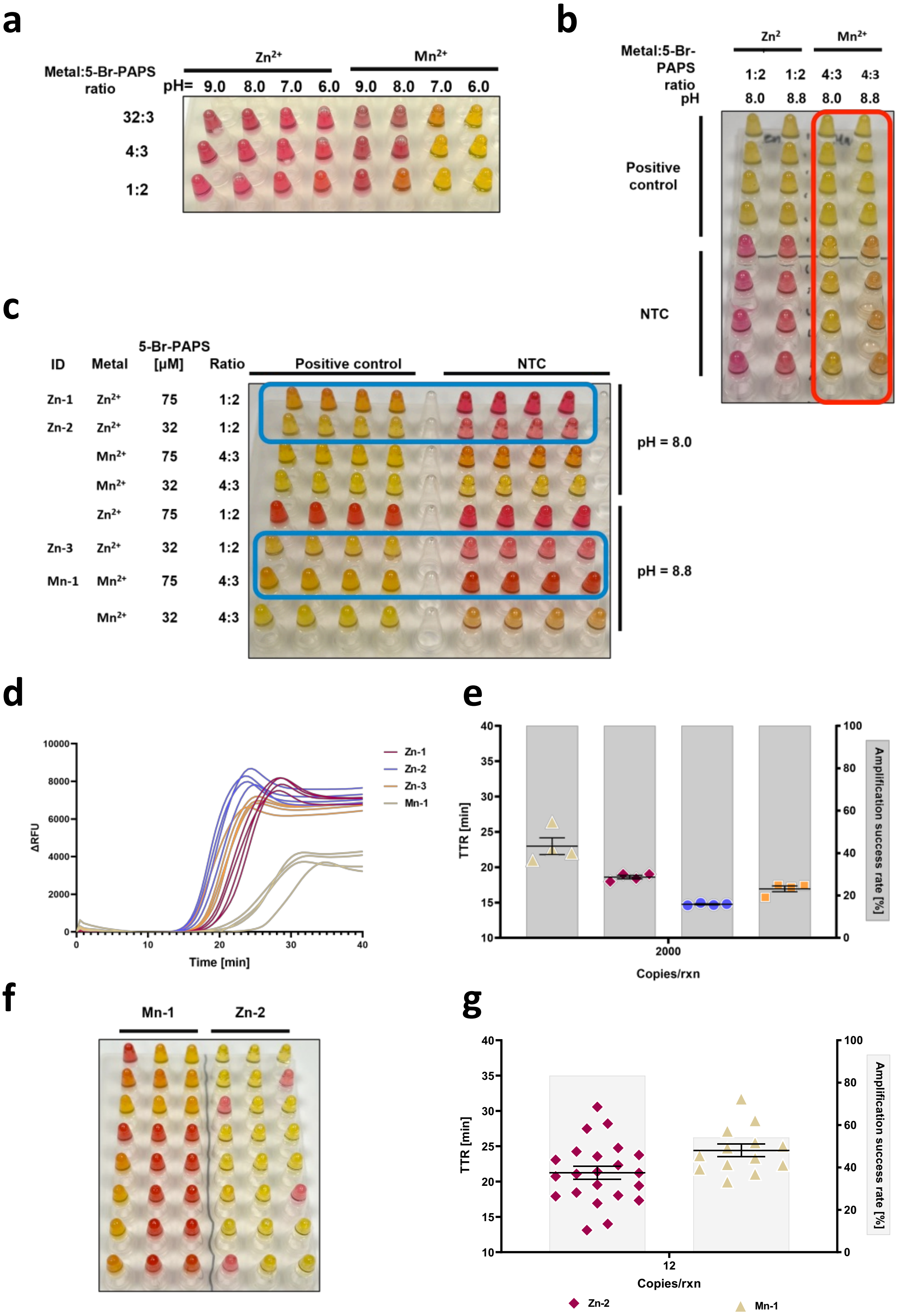

Supplementary Fig. 8. Comparison of Zn^{2+} and Mn^{2+}-based colorimetric detection in RT-LAMP.

a Dependence of 5-Br-PAPS complex formation on metal concentration and pH of Mn^{2+} and Zn^{2+}. Unlike in the case of Zn^{2+}, complex formation of Mn^{2+} with 5-Br-PAPS requires metal excess (4:3 or higher; relative to a normal complex stoichiometry of 1:2) and/or high pH (9.0) to form as evidenced by the gradual shift of color from yellow to purple. All mixes contained 150 mM KCl buffered with 10 mM Tris-HCl (pH=8/9) or MES-NaOH (pH=6/7), 75 µM of 5-Br-PAPS, and specified concentrations of metal ions.

b Comparison of Zn^{2+} and Mn^{2+}-based colorimetric detection in Vivid COVID-19 LAMP master mix base with changes to the pH and metal used. Annotated plate images show colorimetric results of SARS-CoV-2 RNA-spiked (2000 cp/rxn) and NTC reactions performed with either Zn^{2+} or Mn^{2+}-based detection system. Differences in the effects of reaction mix pH and metal:5-Br-PAPS ratio were also explored. 5-Br-PAPS concentration was constant at 32 µM. Mn^{2+} based system failed to function as its starting color was close to the endpoint of the reaction, highlighted in red.

c-e Comparison of Zn^{2+} and Mn^{2+}-based colorimetric detection in NEB WarmStart® LAMP Kit (DNA & RNA) master mix base with pH adjusted with HCl as necessary. The figures depict colorimetric **c** and fluorescent **d, e** results of SARS-CoV-2 RNA-spiked (2000 cp/rxn) and NTC reactions performed with either Zn^{2+} or Mn^{2+}-based detection system. Reactions were supplemented with 40 mM GuCl and 500 nM SYTO 59 and amplified at 65 °C with the quadruplexed primer mix for SARS-CoV-2 detection. Differences in the effects of reaction mix pH, 5-Br-PAPS concentration, and metal:5-Br-PAPS ratio were also explored. Mn^{2+} based system displayed acceptable color transition only at pH 8.8 and with a high excess and concentration of Mn^{2+} (100 µM, 4:3 ratio; Mn-1) in contrast to Zn^{2+}, where the optimal conditions were pH 8.0 and low Zn^{2+} concentration and ratio (16 µM, 1:2 ratio; Zn-2) which corresponds to conditions established for ZBT RT-LAMP. Additionally, Zn^{2+} also performed adequately with high Zn^{2+} concentration at pH=8.0 (37.5 µM, 1:2 ratio; Zn-1) and low Zn^{2+} concentration at pH=8.8 (16 µM, 1:2 ratio; Zn-3). All combinations judged acceptable are highlighted in blue. Importantly, when assessing real time fluorescence, the optimally performing Mn^{2+} master mix displayed impeded reaction speed and lower amplification yield (lower ΔRFU) vs both the optimal and suboptimal Zn^{2+} master mixes. n=4 technical replicates per group were used.

f-g Sensitivity impact of Mn^{2+}/5-Br-PAPS in NEB WarmStart® LAMP Kit (DNA & RNA) master mix base. Direct comparison of Mn-1 and Zn-2 mixes was performed with the same general conditions used in **c-e**, but template input was 12 copies/rxn. Both colorimetric **f** as well as fluorescent **g** data show a significant reduction in sensitivity and reaction speed when using the Mn^{2+}/5-Br-PAPS detector versus the Zn^{2+}/5-Br-PAPS one. n=24 technical replicates per group were used.

The highest time to reaction value on the y-axis equals the total duration of the reaction. Error bars represent standard error of the mean. NTC – no template control; TTR – time to reaction; ΔRFU – normalized relative fluorescence units; cp/rxn – copies per reaction.
