# Supplementary material for: Vivid COVID-19 LAMP is an ultrasensitive, quadruplexed test using LNA-modified primers and a zinc ion and 5-Br-PAPS colorimetric detection system
Source: Commun Biol. 2023 Mar 2;6:233. doi: 10.1038/s42003-023-04612-9 (PMC9979146; doi:10.1038/s42003-023-04612-9)
Supplement: Supplementary file 3 — Description of Additional Supplementary Files [file 42003_2023_4612_MOESM3_ESM.pdf]

Description of Additional Supplementary Files

File name: Supplementary Data 1
Description: Compositions of different LAMP reaction mixes used throughout this study.

File name: Supplementary Data 2
Description: RT-LAMP primer sequences used throughout this study.

File name: Supplementary Data 3
Description: Diagnostic parameters of compared SARS-CoV-2 LAMP assays on low viral load extracted RNA and direct gargle samples.

File name: Supplementary Data 4
Description: : Vivid COVID-19 LAMP In silico cross-reactivity analysis.

File name: Supplementary Data 5
Description: Detailed patient sample information for variant-specific samples used in this study.

File name: Supplementary Data 6
Description: : Diagnostic parameters of ZBP RTLAMP (RNA and direct) and Vivid COVID-19 LAMP.

File name: Supplementary Data 7
Description: Comparison of 10 key features between Vivid COVID-19 LAMP and other state-of-the-art LAMP tests.

File name: Supplementary Data 8
Description: Summarized data of field testing performed with Vivid COVID-19 LAMP in Slovakia.

File name: Supplementary Data 9
Description: Confusion matrices (3x3) with raw numerical data comparing system performance with a human operator.

File name: Supplementary Data 10
Description: Source data behind the graphs in the paper.

File name: Supplementary Video 1
Description: Clip demonstrating software for the reported mobile POCT system.
