# Supplementary material for: Vivid COVID-19 LAMP is an ultrasensitive, quadruplexed test using LNA-modified primers and a zinc ion and 5-Br-PAPS colorimetric detection system
Source: Commun Biol. 2023 Mar 2;6:233. doi: 10.1038/s42003-023-04612-9 (PMC9979146; doi:10.1038/s42003-023-04612-9)
Supplement: Supplementary file 7 — Reporting summary [file 42003_2023_4612_MOESM7_ESM.pdf]

nature portfolio

Corresponding author(s): Evan D. Paul and Pavol Cekan

Last updated by author(s): Feb 3, 2023

Reporting Summary

Nature Portfolio wishes to improve the reproducibility of the work that we publish. This form provides structure for consistency and transparency in reporting. For further information on Nature Portfolio policies, see our Editorial Policies and the Editorial Policy Checklist.

Statistics

For all statistical analyses, confirm that the following items are present in the figure legend, table legend, main text, or Methods section.

n/a	Confirmed	
☐	☒	The exact sample size (*n*) for each experimental group/condition, given as a discrete number and unit of measurement
☐	☒	A statement on whether measurements were taken from distinct samples or whether the same sample was measured repeatedly
☐	☒	The statistical test(s) used AND whether they are one- or two-sided<br>*Only common tests should be described solely by name; describe more complex techniques in the Methods section.*
☒	☐	A description of all covariates tested
☒	☐	A description of any assumptions or corrections, such as tests of normality and adjustment for multiple comparisons
☐	☒	A full description of the statistical parameters including central tendency (e.g. means) or other basic estimates (e.g. regression coefficient) AND variation (e.g. standard deviation) or associated estimates of uncertainty (e.g. confidence intervals)
☒	☐	For null hypothesis testing, the test statistic (e.g. *F*, *t*, *r*) with confidence intervals, effect sizes, degrees of freedom and *P* value noted<br>*Give P values as exact values whenever suitable.*
☒	☐	For Bayesian analysis, information on the choice of priors and Markov chain Monte Carlo settings
☒	☐	For hierarchical and complex designs, identification of the appropriate level for tests and full reporting of outcomes
☒	☐	Estimates of effect sizes (e.g. Cohen's *d*, Pearson's *r*), indicating how they were calculated

Our web collection on statistics for biologists contains articles on many of the points above.

Software and code

Policy information about availability of computer code

Data collection	The source code, input image files and ground truth human classifications, executable Windows Powershell script, and readme.txt (instructions for use) pertaining to the machine-guided software for classification of colorimetric reaction results can be downloaded freely on GitHub (https://github.com/MultiplexDX/LAMP-extractor/tree/main/lamp-extractor).
Data analysis	All data was analyzed using Microsoft Excel or GraphPad Prism 9.0.

For manuscripts utilizing custom algorithms or software that are central to the research but not yet described in published literature, software must be made available to editors and reviewers. We strongly encourage code deposition in a community repository (e.g. GitHub). See the Nature Portfolio guidelines for submitting code & software for further information.

Data

Policy information about availability of data

All manuscripts must include a data availability statement. This statement should provide the following information, where applicable:

- Accession codes, unique identifiers, or web links for publicly available datasets
- A description of any restrictions on data availability
- For clinical datasets or third party data, please ensure that the statement adheres to our policy

The data that support the findings of this study are available within the paper and its supplementary information files. The source data used to construct the figures

and tables is provided in Supplementary Data 1 and 2, respectively. The underlying data may also be obtained from the corresponding authors upon reasonable request.

Human research participants

Policy information about studies involving human research participants and Sex and Gender in Research.

Reporting on sex and gender	Although samples from both males and females of various ages were collected and evaluated, this information was not provided to us due to privacy protection regulations. Therefore, this information was not used for study design or analysis of the data. Since there is no evidence that sex, gender, or age influence the results of nucleic acid tests such as RT-LAMP or RT-qPCR, this should not affect the results of the study.
Population characteristics	See above.
Recruitment	The specimens obtained from participants were part of routine testing by the Biomedical research Center of the Slovak Academy of Sciences and therefore were not subject to any recruiting inclusion or exclusion criteria. Thus, enrollment of participants and selection of specimens was inherently random and not subject to biases that could impact the results.
Ethics oversight	The Ethics committee of the Biomedical research Center of the Slovak Academy of Sciences, Bratislava, Slovakia (Ethics committee statement No. EK/BmV-02/2020)

Note that full information on the approval of the study protocol must also be provided in the manuscript.

Field-specific reporting

Please select the one below that is the best fit for your research. If you are not sure, read the appropriate sections before making your selection.

☒ Life sciences ☐ Behavioural & social sciences ☐ Ecological, evolutionary & environmental sciences

For a reference copy of the document with all sections, see nature.com/documents/nr-reporting-summary-flat.pdf

Life sciences study design

All studies must disclose on these points even when the disclosure is negative.

Sample size	Minimum samples sizes were determined using European Commission guidelines for obtaining CE-marked in vitro diagnostic approval for SARS-CoV-2 nucleic acid tests, which require at least 30 positive and 30 negative specimens. In addition, since our samples were collected as a part of routine testing, our final sample sizes and distribution of positive and negative samples were determined by availability of freshly collected specimens as well as SARS-CoV-2 prevalence during the given time period. Total clinical sample numbers for each clinical validation are as follows:<br><br>RNA SARS-CoV-2 ZBP RT-LAMP: 139 SARS-CoV-2 positive samples (81 NP swabs eluted in CDC viral transport medium (VTM) and 58 isotonic saline gargle samples) and 111 negative samples (48 NP swabs eluted in CDC VTM and 63 isotonic saline gargle samples).<br><br>Direct SARS-CoV-2 ZBP RT-LAMP: 75 SARS-CoV-2 positive samples (43 NP swabs eluted in CDC VTM and 32 isotonic saline gargle samples) and 56 negative samples (24 NP swabs eluted in CDC VTM and 32 isotonic saline gargle samples).<br><br>Vivid COVID-19 LAMP: 108 SARS-CoV-2 positive and 65 negative samples all of which were isotonic saline gargle samples.<br><br>Benchmarking of Vivid COVID-19 LAMP test to other state-of-the-art LAMP tests: 48 SARS-CoV-2 positive and 32 negative samples all of which were isotonic saline gargle samples.
Data exclusions	No data was excluded from the study.
Replication	Multiple technical replicates were utilized to ensure reproducibility and exact numbers are listed in the methods, results, and figures/figure legends.
Randomization	Specimens used in the study were not selected according to any predefined criteria and were obtained as part of a routine SARS-CoV-2 testing program so they are inherently random. Also, since we did not assign samples to experimental groups, randomization is not relevant to this study.
Blinding	All clinical validations were performed by a blinded experimenter at Biomedical Research Center, Institute of Virology, Slovak Academy of Sciences (BMC-SAS) and both the index and evaluated tests were performed at the same time minimizing any possible evaluation bias.

Reporting for specific materials, systems and methods

We require information from authors about some types of materials, experimental systems and methods used in many studies. Here, indicate whether each material, system or method listed is relevant to your study. If you are not sure if a list item applies to your research, read the appropriate section before selecting a response.

Materials & experimental systems

n/a	Involved in the study
☒	☐ Antibodies
☒	☐ Eukaryotic cell lines
☒	☐ Palaeontology and archaeology
☒	☐ Animals and other organisms
☒	☐ Clinical data
☒	☐ Dual use research of concern

Methods

n/a	Involved in the study
☒	☐ ChIP-seq
☒	☐ Flow cytometry
☒	☐ MRI-based neuroimaging
